# Artificial intelligence-aided detection for prostate cancer with multimodal routine health check-up data: an Asian multi-center study

**DOI:** 10.1097/JS9.0000000000000862

**Published:** 2023-11-20

**Authors:** Zijian Song, Wei Zhang, Qingchao Jiang, Longxin Deng, Le Du, Weiming Mou, Yancheng Lai, Wenhui Zhang, Yang Yang, Jasmine Lim, Kang Liu, Jae Young Park, Chi-Fai Ng, Teng Aik Ong, Qiang Wei, Lei Li, Xuedong Wei, Ming Chen, Zhixing Cao, Fubo Wang, Rui Chen

**Affiliations:** a Department of Urology, Shanghai Changhai Hospital, Second Military Medical University; b Department of Urology, Renji Hospital, Shanghai Jiao Tong University School of Medicine; c Key Laboratory of Smart Manufacturing in Energy Chemical Process, Ministry of Education; d State Key Laboratory of Bioreactor Engineering, East China University of Science and Technology, Shanghai; e Department of Urology, Shanghai General Hospital, Shanghai Jiao Tong University School of Medicine; f Department of Clinical Laboratory, Nanjing Jinling Hospital, Nanjing University School of Medicine; g Department of Urology, University of Malaya Medical Centre, Kuala Lumpur, Malaysia; h SH Ho Urology Centre, Department of Surgery, The Chinese University of Hong Kong, Hong Kong SAR, China; i Department of Urology, Korea University Ansan Hospital, Soule, Korea; j Department of Urology, Institute of Urology, West China Hospital, Sichuan University, Chengdu, Sichuan; k Department of Urology, The First Affiliated Hospital of Xi'an Jiaotong University, Xi'an Shaanxi; l Department of Urology, The First Affiliated Hospital of Soochow University, Suzhou; m Department of Urology, Zhongda Hospital, Southeast University, Nanjing; n School of Life Sciences, Guangxi Medical University, Nanning, Guangxi; o Center for Genomic and Personalized Medicine, Guangxi Key Laboratory for Genomic and Personalized Medicine, Guangxi Collaborative Innovation Center for Genomic and Personalized Medicine, Guangxi Medical University, Nanning, Guangxi; p Department of Urology, the First Affiliated Hospital of Guangxi Medical University, Guangxi Medical University, Guangxi China

**Keywords:** artificial intelligence, diagnosis, prostate biopsy, Prostate cancer, risk prediction

## Abstract

**Background:**

The early detection of high-grade prostate cancer (HGPCa) is of great importance. However, the current detection strategies result in a high rate of negative biopsies and high medical costs. In this study, the authors aimed to establish an Asian Prostate Cancer Artificial intelligence (APCA) score with no extra cost other than routine health check-ups to predict the risk of HGPCa.

**Patients and methods:**

A total of 7476 patients with routine health check-up data who underwent prostate biopsies from January 2008 to December 2021 in eight referral centres in Asia were screened. After data pre-processing and cleaning, 5037 patients and 117 features were analyzed. Seven AI-based algorithms were tested for feature selection and seven AI-based algorithms were tested for classification, with the best combination applied for model construction. The APAC score was established in the CH cohort and validated in a multi-centre cohort and in each validation cohort to evaluate its generalizability in different Asian regions. The performance of the models was evaluated using area under the receiver operating characteristic curve (ROC), calibration plot, and decision curve analyses.

**Results:**

Eighteen features were involved in the APCA score predicting HGPCa, with some of these markers not previously used in prostate cancer diagnosis. The area under the curve (AUC) was 0.76 (95% CI:0.74–0.78) in the multi-centre validation cohort and the increment of AUC (APCA vs. PSA) was 0.16 (95% CI:0.13–0.20). The calibration plots yielded a high degree of coherence and the decision curve analysis yielded a higher net clinical benefit. Applying the APCA score could reduce unnecessary biopsies by 20.2% and 38.4%, at the risk of missing 5.0% and 10.0% of HGPCa cases in the multi-centre validation cohort, respectively.

**Conclusions:**

The APCA score based on routine health check-ups could reduce unnecessary prostate biopsies without additional examinations in Asian populations. Further prospective population-based studies are warranted to confirm these results.

## Introduction

Highlights

- The AI-based models with routine health check-up data can reduce unnecessary prostate biopsies.

Prostate cancer (PCa) is the first leading malignancy and the second leading cause of death in males worldwide^1^ and has become rapidly prevalent among Asian men in recent years^2^. High-grade PCa (HGPCa) refers to International Society of Urological Pathology (ISUP) grade 2 or higher. HGPCa is associated with a poor prognosis, whereas low-grade PCa (LGPCa) is often indolent and non-lethal. Thus, the current diagnostic strategy for PCa focuses on HGPCa detection. The introduction of prostate-specific antigen (PSA) has greatly improved the detection of PCa, but it has been criticized for its low specificity. For instance, biopsies may be unnecessary for a high proportion of patients with PSA 4–20 ng/ml in Asian populations, thereby incurring pain for patients and imposing burdens on the healthcare system. Although traditional risk prediction models, novel biomarkers, and multiparameter MRI (mpMRI) have been reported to substantially increase diagnostic accuracy, they suffer from several drawbacks. Risk prediction models such as the ERSPC Risk Calculator 4 and the Chinese Prostate Cancer Consortium Risk Calculator^3^ depend on specialized urological examinations, including digital rectal examinations and transrectal ultrasonography, which are rarely involved in general health check-ups^4^. Biomarkers (p2PSA, PCA3, 4Kscore, etc.)^5^ and mpMRI were helpful in improving the diagnostic accuracy in patients at high risk of prostate cancer, but they were associated with high cost and low availability in health check-ups or pre-screening settings. We intended to establish a prediction tool with multimodal routine health check-up data to identify HGPCa, differentiate men with the need for prostate biopsies, and thereby help democratize prostate cancer screening.

Artificial intelligence (AI), typically machine learning, is excellent at making use of computational power to distil quantitative representations between a vast number of predictors and outcomes, and its efficacy has been evidenced by massive success in clinics^6,7^. The emergence of large language models such as ChatGPT can answer the questions and concerns of patients with prostate cancer and help democratize medical knowledge^8^. However, few studies have involved parameters in routine health check-ups, such as routine blood tests, blood biochemistry tests, routine urine tests, and abdominal ultrasound. These tests include a large number of predictors, commonly neglected in predicting PCa, that could be combined by AI technology, as shown in some pioneering studies on lung cancer^6^ and breast cancer^7^. In this study, we established the Asian Prostate Cancer AI Score (APCA Score) for predicting HGPCa with independent external validation in seven Asian urological centres with distinctive geographic and clinical characteristics. Validating the APCA Score in an individual centre highlights its high generalization ability in an actual clinical scenario.

## Materials and methods

### Patient selection

The study protocol was approved by the Institutional Ethics Committee of CH and registered in the Clinical Trial Registry. This retrospective study was performed according to the TRIPOD guidelines, Supplemental Digital Content 1, http://links.lww.com/JS9/B363 and STARD (Standards for the Reporting of Diagnostic accuracy studies) criteria^9^, Supplemental Digital Content 2, http://links.lww.com/JS9/B364. However, the data were collected consecutively and prospectively, which can help ascertain the chronological order of causality and reduce the proportion of missing data to a certain extent. Multi-centre data were collected from patients who underwent prostate biopsy at Shanghai Changhai Hospital (CH), Zhongda Hospital (ZH), the First Affiliated Hospital of Soochow University (SU), West China Hospital (WCH), the First Affiliated Hospital of Xi'an Jiaotong University (XAJU) in Chinese mainland, Korea University Ansan Hospital (AH) in South Korea, the Prince of Wales Hospital of The Chinese University of Hong Kong (PWH) in Hong Kong, and the University of Malaya Medical Centre (UM) in Malaysia. Patients with indications for prostate biopsy according to the international guidelines were included. Health check-up data were collected within six months before prostate biopsy. We included patients with PSA between 4.0 and 20.0 ng/ml rather than only men with PSA 10.0 ng/ml, based on previous findings that Asian men have a lower PCa detection rate in the same PSA range compared to Caucasian men^10^, which was supported by a recent study^11^. Exclusion criteria were previous prostate biopsy, previous treatment of PCa, and previous surgical intervention of the prostate.

### Data Pre-processing

**Figure 1:**
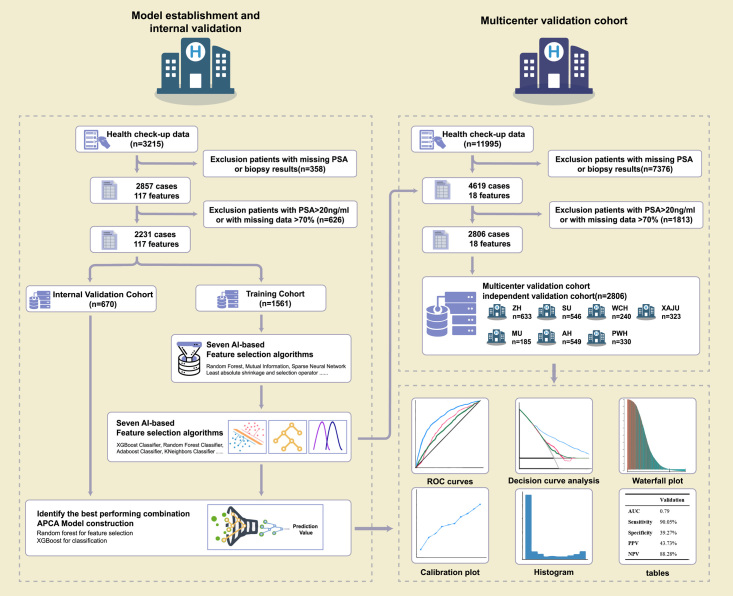
The workflow of this study and workflow the data analysis.

The study design is summarized in Figure 1. A total of 15 210 anonymized prospectively collected medical records of routine health check-up data (blood routine tests, blood biochemistry tests, urine routine tests, abdominal ultra-sounds, etc.) were retrieved for patients who underwent an initial prostate biopsy from the Hospital Information System (HIS) of eight participating hospitals. A total of 7476 patients who underwent prostate biopsies between January 2008 and December 2021 were included after initial data screening for essential clinical information (pathological results and PSA level). Data cleaning Quality control was performed for all laboratory features. Data cleaning was applied, including transferring medical symbols to digital symbols, deleting cases with information completeness less than 60% (indicating patients with limited available features), deleting features with information completeness less than 60% (indicating features that were not widely tested), and deleting men with a higher and lower PSA level (PSA 20 ng/ml or higher, indicating a high likelihood of PCa, also PSA 4 ng/ml or lower, considering biopsy for reasons other than elevated PSA, which are beyond the scope of this study). After data cleaning, 117 clinical and laboratory features were included (Supplementary Table 1, Supplemental Digital Content 2, Supplemental Digital Content 3, http://links.lww.com/JS9/B365). A total of 5037 cases were involved, including 2231 from CH, 549 from AH, 633 from ZH, 546 from SU, 330 from PWH, 323 from XAJU, 240 from WCH, and 185 from MU. We included much more participants than the minimal required sample size calculated with PASS software. The APAC score was established in the CH cohort and validated in a multi-centre cohort and in each validation cohort to evaluate its generalizability in different Asian regions. Missing data imputation: For qualitative data, the mode number was used to fill in missing values, whereas for quantitative data, the mean was used to fill in missing values.

### AI-based algorithm selection

Feature selection is applied to reduce the number of features to be tested using different classifiers. The CH cohort was used in the feature-selection process by randomly splitting the data into training and validation cohorts (7:3). Seven feature selection algorithms were applied, including Random Forest, XGBoost and Sparse Neural Network, etc^12^. For model development, seven classification algorithms were applied, including the XGBoost classifier, random forest classifier, Adaboost classifier, KNeighbors classifier, multilayer perceptron classifier, support vector machine classifier, and decision tree classifier. We tested 49 possible combinations of feature selection and classification algorithms to identify the best-performing combination. The best-performing combination of feature selection and classification was selected to establish the APCA score.

### Model establishment and validation

The APCA score was established in 70.0% of the randomly selected men from the CH cohort and was integrally validated in the remaining 30.0% of the men from the CH cohort. Binary cross-entropy was used as the loss function, and the gradient-boosting algorithm was used to optimize the model. The hyperparameters of the APCA score were as follows: learning rate=0.3, N-estimators=100, Max-depth=6, Sub-sample=1, and Colsample-bytree, 1. The APCA score was externally validated in seven individual cohorts and multi-centre cohort to test the performance of the prediction model.

### Statistical analysis

The receiver operating characteristic curve (ROC) was applied to evaluate model discrimination, and the area under the curve (AUC) was used to compare diagnostic performance. Decision curve analysis (DCA), as described by Vickers and Elkin^13^, was performed to assess the clinical application performance of the APCA score, PSA, and fPSA/PSA by quantifying the net benefits at a spectrum of threshold probabilities. Calibration curves were used to assess the agreement between the actual and predicted HGPCa. As a pre-screening model, the threshold of the APCA score was set at a sensitivity of approximately 90.0% to avoid missing HGPCa. Subsequently, the specificity, positive predictive value (PPV), and negative predictive value (NPV) were calculated. Data pre-processing, machine-learning model development, and validation were conducted with Python 3.2 and the R package version 3.5.3 (www.r-project.org).

## Results

### Patient characteristics and the AI-based algorithms

**Figure 2:**
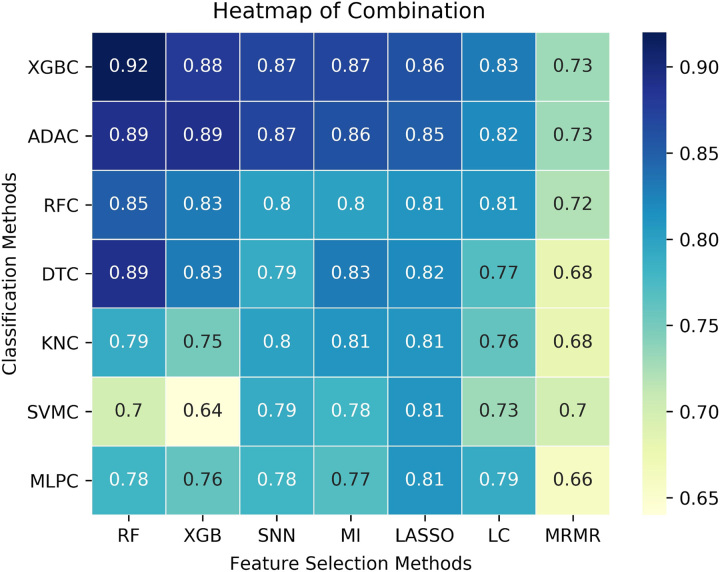
Heat map shows the area under the receiver operating characteristic curve (AUC) suggesting diagnostic performance of 49 classification algorithm (rows) and feature-selection algorithm (columns) combinations applied to validation set. ADAC,= Adaboost Classifier; DTC, Decision Tree Classifier; KNC, KNeighbors Classifier; Lasso, Least absolute shrinkage and selection operator; LC, linear correlation; MI, mutual information; MLPC, Multilayer Perceptron Classifier; MRMR, max-relevance and min-redundancy; RF, Random Forest; RFC, Random Forest Classifier; SNN, Sparse Neural Network; SVMC, Support Vector Machines Classifier; XGB, XGBoost; XGBC, XGBoost Classifier.

A total of 5037 patients and 117 features were finally analyzed in this study. The characteristics of the included patients are summarized in Table 1. In brief, 1677 (32.3%) and 3515 (67.7%) participants were pathologically diagnosed with HGPCa and non-HGPCa (negative biopsy or LGPCa), respectively. The percentage of PCa ranged from 24.6% to 53.9% among the eight participating hospitals. The combination of Random Forest for feature selection and XGBoost for classification had the highest AUC (0.93 in the CH internal validation cohort among the 49 combinations (Fig. 2). The features included in the APCA score are listed in Supplementary Table S2 in Supplemental Digital Content, Supplemental Digital Content 3, http://links.lww.com/JS9/B365.

**Table 1:** Patients’ characteristics in different cohorts.

Features (Media, IQR)	CH Development	CH Validation	Multi-centre cohort	ZH	SU	WCH	XAJU	PWH	AH	MU
No. pts, *n* (%)	1561 (30.9)	670 (13.3)	3476 (69.1)	633 (12.5)	546 (10.8)	240 (4.7)	323 (6.4)	330 (6.5)	549 (10.8)	185 (3.6)
Age (Media, IQR)	68 (62–73)	67 (62–73)	68 (62–74)	69 (63–76)	67 (61–74)	67 (61–73)	69 (64.5–75)	69 (65–73)	66 (60–73)	70 (65–74)
Any PCa (%)	39.1%	39.2%	35.3%	24.6%	34.7%	30.4%	21.6%	53.9%	43.5%	31.8%
HGPCa (%)	29.7%	29.7%	25.3%	19.4%	28.7%	25.4%	19.5%	29.6%	24.5%	23.7%
PSA (ng/ml)	9.0 (6.7–12.3)	8.9 (6.6–11.5)	8.9 (6.5–12.5)	8.8 (6.3–12.7)	9.6 (7.1–13.1)	10.4 (7.3–13.8)	10.9 (7.9–14.2)	8.7 (6.4–11.6)	7.4 (5.5–10.3)	8.4 (6.4–11.2)
fPSA (ng/ml)	1.30 (0.83–1.86)	1.30 (0.84–1.93)	1.33 (0.87–1.88)	1.35 (0.96–1.93)	1.26 (0.76–1.94)	1.26 (0.83–2.04)	1.52 (1.06–2.38)	1.40 (1.22–1.40)	1.02 (0.71–1.52)	NA
fPSA/PSA	0.15 (0.10–0.20)	0.15 (0.10–0.21)	0.15 (0.10–0.20)	0.16 (0.11–0.21)	0.13 (0.09–0.19)	0.14 (0.10–0.19)	0.15 (0.11–0.2)	0.18 (0.17–0.18)	0.14 (0.10–0.19)	NA
LR*UL (cm2)	20.2 (18.6–25.0)	20.7 (18.6–25.0)	21.3 (17.6–25.4)	25.0 (18.6–28.1)	21.5 (17.3–25.5)	21.3 (17.3–25.6)	25.0 (20.3–29.7)	24.9 (19.8–28.8)	17.4 (14.5–21.2)	20.8 (18.9–20.8)
Urinary specific gravity (%)	1.016 (1.010–1.021)	1.025 (1.010–1.020)	1.016 (1.012–1.020)	1.018 (1.015–1.025)	1.016 (1.010–1.020)	NA	1.016 (1.012–1.019)	NA	1.017 (1.012–1.021)	1.013 (1.01–1.016)
Serum alkaline phosphatase (u/l)	78.1 (66.0–89.0)	78.1 (67.0–89.0)	73.0 (60.0–84.0)	69.0 (57.0–83.0)	72.3 (59.3–85.4)	NA	78.1 (67.0–92.0)	NA	68.0 (57.0–77.0)	71.0 (60.0–83.0)
Serum albumin (g/l)	41.9 (40.0–44.0)	41.9 (40.0–43.0)	41.9 (38.8–44.9)	40.5 (37.8–43.2)	43.1 (40.3–46.1)	NA	43 (39.5–45.8)	38.7 (37.2–40.6)	44.0 (42.0–46.0)	38.0 (35.0–40.0)
Blood glucose (mmol/l)	6.0 (5.3–6.4)	6.0 (5.4–6.3)	5.6 (5.1–6.1)	5.7 (5.1–6.8)	5.2 (4.8–5.7)	NA	5.2 (4.7–6.0)	5.9 (5.3–6.1)	NA	5.7 (5.3–6.1)
Lymphocyte percentage (%)	26.6 (21.1–31.8)	27.7 (21.2–32.5)	26.5 (21.1–32.5)	24.8 (17.8–31.1)	28.5 (22.9–33.5)	27.5 (21.3–33.2)	24.0 (18.3–29.1)	26.4 (25.8–28.3)	30.1 (24.0–35.9)	22.8 (18.4–27.5)
Serum potassium (mmol/l)	4.0 (3.9–4.2)	4.0 (3.9–4.2)	4.1 (3.9–4.4)	3.9 (3.7–4.1)	4.1 (3.8–4.3)	NA	4.1 (3.9–4.3)	4.2 (4.0–4.4)	4.4 (4.1–4.6)	4.2 (4–4.5)
Serum sodium (mmol/l)	141.4 (141.0–143.0)	141.4 (141.0–143.0)	141.0 (139.6–142.6)	140.0 (138.4–141.7)	141.5 (140.2–142.9)	NA	142.0 (140.0–144.0)	141.0 (139.7–142.3)	141.0 (140.0–142.0)	139.0 (137.0–140.0)
Platelet distribution width (fl)	13.5 (11.6–16.0)	13.3 (11.6–16.0)	13.7 (11.9–16)	13.4 (11.8–15.8)	13.7 (11.9–16.1)	15.0 (13.5–16.5)	13.8 (12.2–16.1)	NA	NA	NA
Blood haematocrit (%)	43.3 (40.6–45.6)	43.3 (40.7–45.3)	41.3 (36.0–44.4)	41.0 (37.5–43.6)	43.0 (40.0–45.8)	44.0 (42.0–46.0)	43.1 (39.7–46.0)	41.9 (40.0–44.1)	42.3 (40.0–44.6)	41.0 (38.0–44.0)
Gamma-Glutamyl transpeptidase (u/l)	33.9 (28.0–34.1)	33.9 (33.2–34.1)	28.0 (19.0–34.1)	25.0 (18.0–36.0)	23.2 (17.1–36.1)	NA	24.0 (16.0–34.0)	NA	NA	27.0 (19.0–35.0)
Blood lymphocyte count (%)	1.6 (1.2–2.0)	1.6 (1.3–2.1)	1.7 (1.3–2.3)	1.5 (1.1–1.9)	1.6 (1.2–2.0)	1.7 (1.4–2.0)	1.4 (1.1–1.8)	1.7 (1.5–1.8)	3.6 (2.8–4.6)	1.7 (1.4–2.0)
Blood neutrophil count (10^9/l)	3.8 (3.0–4.8)	3.8 (3.0–4.9)	3.6 (2.7–4.7)	4.1 (3.2–5.3)	3.5 (2.8–4.3)	NA	4.2 (3.2–5.3)	4.5 (3.5–4.5)	1.9 (1.5–2.3)	4.9 (4.0–6.5)
Blood mean corpuscular haemoglobin (pg)	30.8 (30.0–31.6)	30.8 (29.9–31.6)	30.7 (29.7–31.7)	30.6 (29.7–31.5)	30.7 (29.8–31.7)	30.7 (29.6–31.6)	31.1 (30.2–32.1)	30.8 (29.8–32.0)	30.8 (30.0–31.8)	29.3 (28.0–30.4)

fPSA, free prostate-specific antigen; HGPCa, high-grade prostate cancer; LR*UL, ultrasound left and right diameter *upper and lower diameter; NA, not applicabler; PCa, prostate cancer; PSA, prostate-specific antigen; Pts, patients.

### Diagnostic performance of the APCA score

**Figure 3:**
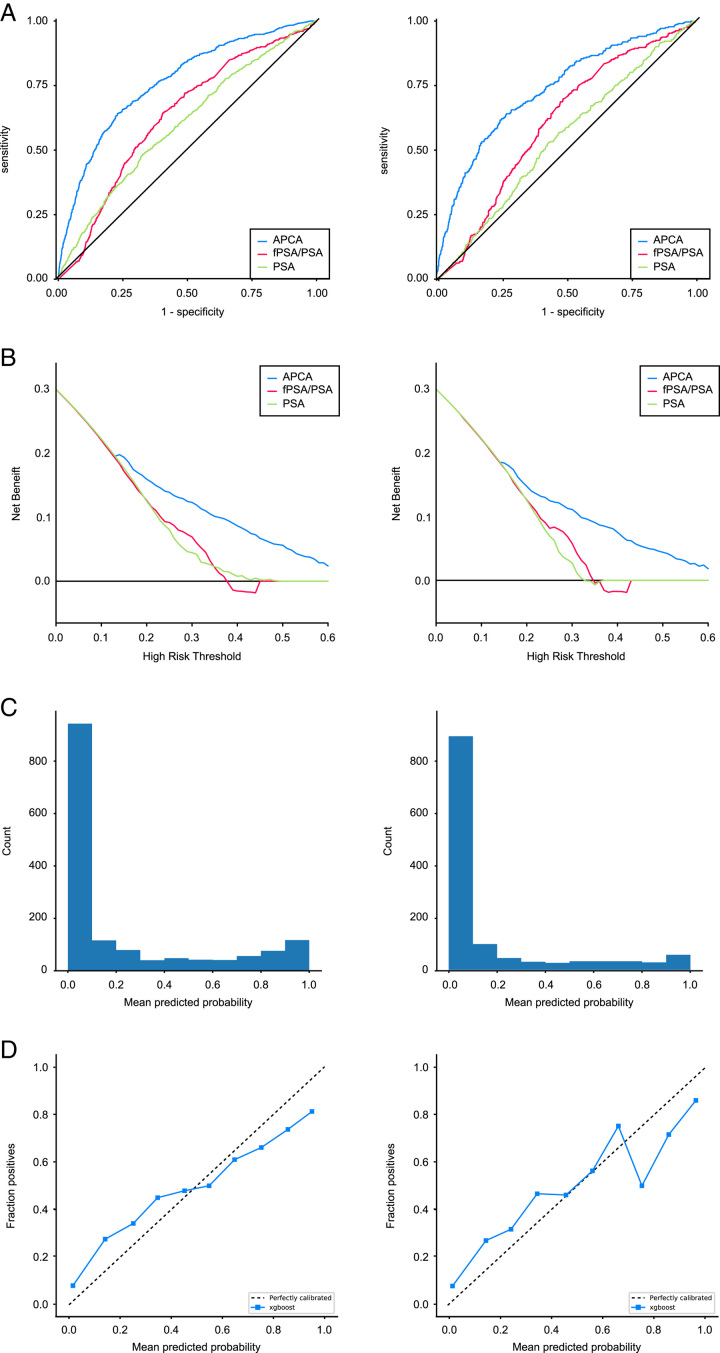
Receiver operating characteristic (ROC) curves, calibration plot, and decision curve analysis of the APCA Score in predicting HGPCa in men with varying PSA levels. [(A) ROC curves, (B) Calibration plot, (C) Histogram of mean predicted possibility, (D) Decision curve analysis] with PSA 4–20 ng/ml represented on the left panels and PSA 4–10 ng/ml on the right panels.

**Figure 4:**
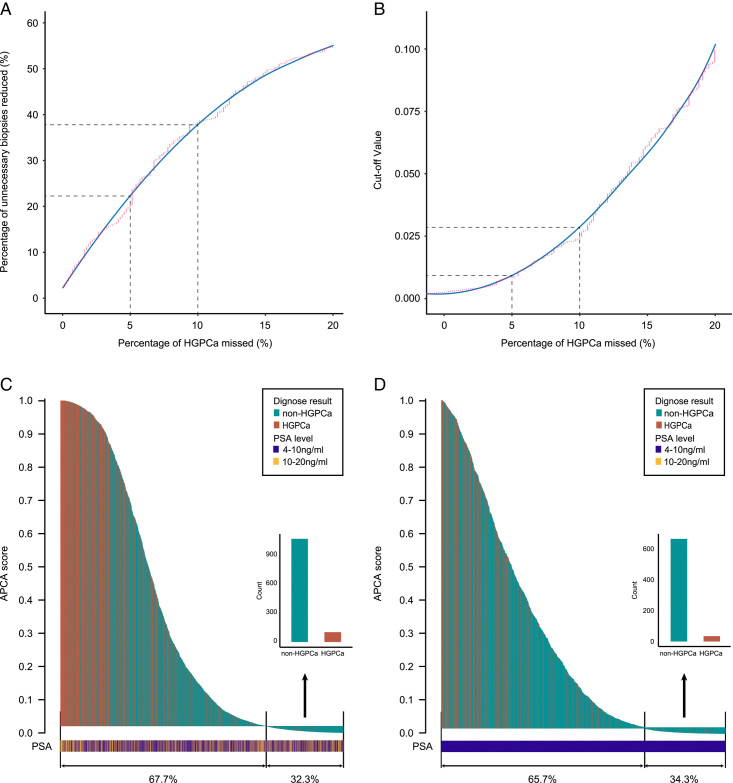
Reduction of unnecessary biopsies and the corresponding risk of missing HGPCa at different cutoff values. (A) Percentage of HGPCa missed and percentage of unnecessary biopsies reduced with different cutoff values (B) Percentage of HGPCa missed and corresponding APCA score cutoff value. The red points represent the percentage of HGPCa missed and the percentage of unnecessary biopsies reduced, and the blue curve is the loess-smoothed fitted curve. (C) Waterfall plot of the APCA score related to prostate biopsy results in the multi-centre validation cohort for (C) PSA 4–20 ng/ml and (D) PSA 4–10 ng/ml. (C, D) Each bar represents a single individual. Red indicates HGPCa; blue indicates non-HGPCa. The horizontal lines represent the cutoff points of 0.02012 and 0.01625 at a sensitivity of nearly 90.0% in men with PSA 4–20 ng/ml and 4–10 ng/ml, respectively.

In men with PSA 4–20 mg/ml, 2226 men were predicted as HGPCa with HGPCa in 806 cases and non-HGPCa in 1420 cases, while 1250 men were predicted as non-HGPCa with HGPCa in 74 cases and non-HGPCa in 1176 cases. The APCA score yielded high predictive accuracy for the prediction of HGPCa in men with PSA 4–20 mg/ml, with an AUC of 0.93 in the internal validation and 0.76 (95% CI:0.74–0.78) in the multi-centre validation cohort. The AUC was 0.83 (95% CI:0.79–0.87), 0.77 (95% CI:0.73–0.82), 0.75 (95% CI:0.68–0.82) 0.88 (95% CI:0.83–0.93) 0.72 (95% CI:0.65–0.78), 0.72 (95% CI:0.67–0.77) and 0.76 (95% CI:0.68–0.85) in the seven independent validation cohorts, respectively (Table 2, Supplementary Figs S1–2 and Supplementary Table S3 in Supplemental Digital Content 2, Supplemental Digital Content 3, http://links.lww.com/JS9/B365). Although the absolute AUC of the APCA score was not very high in all cohorts, the increment of the APCA score compared with PSA was 0.16 (95% CI:0.13–0.20) in the multi-centre validation cohort and 0.18(95% CI:0.11–0.24), 0.17(95% CI:0.10–0.24), 0.21(95% CI:0.10–0.31), 0.36(95% CI:0.28–0.45), 0.16(95% CI:0.07–0.25), 0.08(95% CI:0.01–0.15), 0.11(95% CI: −0.02 to 0.24) in the seven independent validation cohorts. Meanwhile, for a fixed sensitivity of nearly 90.0%, the specificity was 84.50% in the CH internal validation cohort; 48.11% in the multi-centre validation cohort; and 50.4%, 42.7%, 31.8%, 71.5%, 22.0%, 42.3%, and 34.0% in the seven independent validation cohorts, respectively. The predictive performance of each predictor and the APCA score are summarized (Table 3 and Supplementary Table S4 in Supplemental Digital Content 2, Supplemental Digital Content 3, http://links.lww.com/JS9/B365). The ROC curves, calibration plot, and DCA of the APCA score showed a high performance in predicting HGPCa (Fig. 3) and any PCa in all cohorts (Supplementary Figure S3, Supplemental Digital Content 2, Supplemental Digital Content 3, http://links.lww.com/JS9/B365). The distribution of the APCA score was depicted by a waterfall plot with a cutoff value of nearly 90.0% sensitivity (Fig. 4, Supplementary Fig 4 in Supplemental Digital Content 2, Supplemental Digital Content 3, http://links.lww.com/JS9/B365). The bar chart in Figure 4 showed the biopsy results of patients predicted as non-HGPCa by the APCA score, which indicated that most patients predicted as non-HGPCa by the APCA score could spare a biopsy. The panel below the waterfall plot reflects the PSA level of each patient. Patients with PSA 4.0–10.0 ng/ml and 10.0–20.0 ng/ml were basically distributed evenly across the APCA score from 0.0 to 1.0.

**Table 2:** The AUCs of receiver operator characteristic curves, sensitivity, specificity, positive predictive value, negative predictive value of the APCA score when predicting HGPCa.

	CH Validation	Multi-centre validation cohort	ZH	SU	WCH	XAJU	PWH	AH	MU
PSA 4–10 ng/ml									
AUC	0.93	0.79	0.82	0.78	0.73	0.83	0.65	0.73	0.77
Sensitivity	90.91%	90.05%	90.00%	90.63%	92.86%	92.00%	91.07%	90.12%	90.48%
Specificity	83.39%	39.27%	48.13%	52.89%	15.91%	57.14%	16.23%	43.30%	36.36%
PPV	61.54%	43.73%	21.33%	35.37%	26.00%	32.39%	28.33%	28.63%	23.17%
NPV	96.91%	88.28%	96.86%	95.20%	87.50%	96.97%	83.33%	94.56%	94.74%
PSA 4–20 ng/ml									
AUC	0.93	0.76	0.83	0.77	0.75	0.88	0.72	0.72	0.76
Sensitivity	90.45%	90.01%	90.24%	90.45%	90.16%	90.48%	90.82%	90.37%	90.91%
Specificity	84.50%	38.45%	50.39%	42.67%	31.84%	71.54%	21.98%	42.27%	34.04%
PPV	71.15%	31.91%	30.49%	38.90%	31.07%	43.51%	32.96%	33.80%	30.08%
NPV	95.44%	92.32%	95.54%	91.71%	90.48%	96.88%	85.00%	93.09%	92.31%

APCA, Asian Prostate Cancer AI; AUC, area under the curve; HGPCa, high-grade prostate cancer; NPV, negative predictive value; PPV, positive predictive value; PSA, prostate-specific antigen.

**Table 3:** The AUCs of receiver operator characteristic curves for prediction model and individual predictors when predicting HGPCa at PSA 4–20 ng/ml.

Predictors	CH validation	ZH	SU	WCH	XAJU	PWH	AH	MU
APCA Score	0.93 (0.91–0.95)	0.83 (0.79–0.87)	0.77 (0.73–0.82)	0.75 (0.68–0.82)	0.88 (0.83–0.93)	0.72 (0.65–0.78)	0.72 (0.67–0.77)	0.76 (0.68–0.85)
LR*UL	0.78 (0.74–0.81)	0.78 (0.74–0.83)	0.72 (0.67–0.77)	0.72 (0.64–0.79)	0.85 (0.80–0.91)	0.71 (0.65–0.77)	0.69 (0.64–0.74)	0.72 (0.63–0.81)
SG	0.54 (0.49–0.59)	0.55 (0.49–0.60)	0.52 (0.46–0.57)	NA	0.54 (0.46–0.62)	NA	0.56 (0.50–0.61)	0.56 (0.46–0.65)
ALP	0.49 (0.44–0.54)	0.55 (0.49–0.61)	0.54 (0.49–0.59)	NA	0.52 (0.44–0.60)	NA	0.53 (0.47–0.59)	0.53 (0.43–0.64)
fPSA/PSA	0.71 (0.66–0.75)	0.73 (0.68–0.78)	0.66 (0.61–0.71)	0.68 (0.61–0.75)	0.63 (0.55–0.71)	0.41 (0.34–0.47)	0.60 (0.55–0.66)	NA
ALB	0.50 (0.45–0.55)	0.53 (0.47–0.58)	0.56 (0.50–0.61)	NA	0.54 (0.46–0.62)	0.49 (0.42–0.56)	0.48 (0.43–0.54)	0.50 (0.40–0.60)
fPSA	0.60 (0.56–0.65)	0.57 (0.51–0.63)	0.56 (0.51–0.61)	0.60 (0.52–0.67)	0.60 (0.52–0.68)	0.42 (0.36–0.49)	0.52 (0.47–0.58)	NA
PSA	0.62 (0.57–0.66)	0.65 (0.59–0.70)	0.61 (0.55–0.66)	0.54 (0.46–0.62)	0.51 (0.43–0.60)	0.55 (0.48–0.63)	0.64 (0.58–0.69)	0.65 (0.56–0.75)
Glu	0.48 (0.43–0.53)	0.50 (0.45–0.56)	0.54 (0.49–0.59)	NA	0.59 (0.51–0.67)	0.50 (0.43–0.56)	NA	0.50 (0.41–0.59)
Lym%	0.54 (0.49–0.59)	0.52 (0.47–0.57)	0.54 (0.48–0.59)	0.52 (0.44–0.61)	0.57 (0.49–0.65)	0.52 (0.46–0.59)	0.50 (0.44–0.56)	0.56 (0.46–0.66)
K	0.50 (0.45–0.55)	0.53 (0.47–0.59)	0.51 (0.45–0.56)	NA	0.52 (0.45–0.60)	0.50 (0.43–0.57)	0.55 (0.49–0.60)	0.54 (0.44–0.64)
Na	0.55 (0.50–0.60)	0.51 (0.46–0.57)	0.54 (0.48–0.59)	NA	0.52 (0.44–0.61)	0.50 (0.43–0.57)	0.47 (0.42–0.53)	0.62 (0.52–0.71)
PDW	0.54 (0.50–0.59)	0.52 (0.46–0.58)	0.53 (0.48–0.58)	0.50 (0.41–0.58)	0.55 (0.46–0.63)	NA	NA	NA
HCT	0.48 (0.43–0.52)	0.51 (0.46–0.56)	0.55 (0.50–0.61)	0.55 (0.47–0.64)	0.51 (0.43–0.59)	0.51 (0.44–0.58)	0.55 (0.49–0.60)	0.64 (0.54–0.74)
GGT	0.68 (0.62–0.73)	0.51 (0.45–0.57)	0.54 (0.48–0.59)	NA	0.56 (0.48–0.63)	NA	NA	0.52 (0.42–0.63)
Lym	0.53 (0.49–0.58)	0.48 (0.43–0.54)	0.55 (0.50–0.61)	0.54 (0.45–0.63)	0.58 (0.49–0.66)	0.51 (0.44–0.57)	0.48 (0.43–0.54)	0.47 (0.37–0.57)
Neut	0.51 (0.46–0.55)	0.49 (0.44–0.55)	0.48 (0.43–0.54)	NA	0.53 (0.46–0.61)	0.47 (0.40–0.54)	0.51 (0.45–0.57)	0.44 (0.35–0.54)
MCH	0.54 (0.50–0.59)	0.51 (0.45–0.56)	0.53 (0.48–0.59)	0.56 (0.48–0.64)	0.48 (0.40–0.56)	0.49 (0.42–0.56)	0.50 (0.44–0.56)	0.53 (0.43–0.63)

ALB, serum albumin; ALP, serum alkaline phosphatase; APCAI, Asian Prostate Cancer AI; AUC, area under the curve; fPSA, free prostate-specific antigen; GGT, Gamma-Glutamyl transpeptidase; Glu, blood glucose; Lym%, Llium; LR*UL, ultrasound left and right diameter * upper and lower diameter; Lym, blood lymphocyte count; MCH, blood mean corpuscular haemoglobin; Na, serum sodium; NA, not applicable; Neut, blood neutrophil count; PDW, platelet distribution width; PSA, prostate-specific antigen; SG, urinary specific gravity.

### Predictive performance of individual features

**Figure 5:**
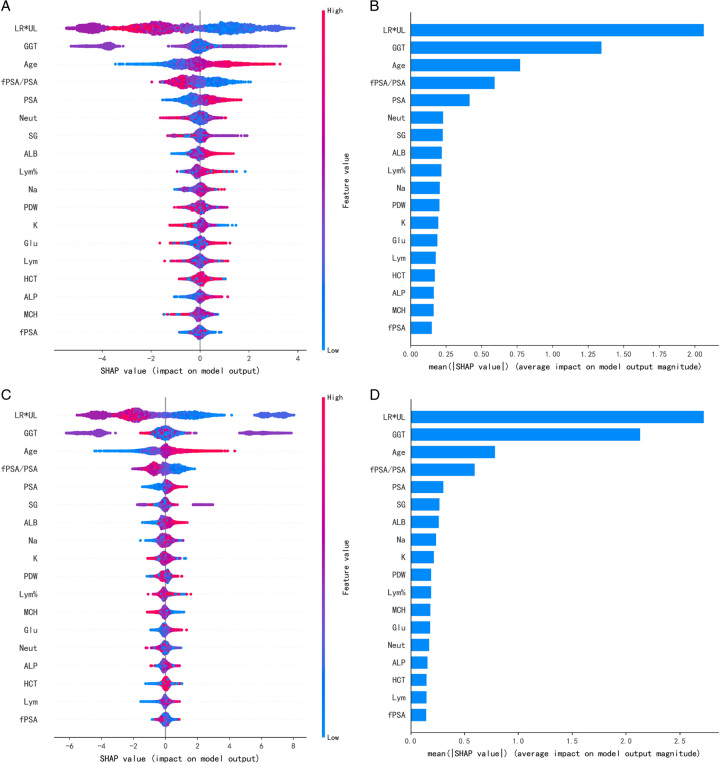
The impact of different features illustrated by SHAP value in HGPCa prediction. The SHAP value plotting the 18 most relevant features to predict the risk of HGPCa [(A) and (B) for PSA 4–20 ng/ml; (C) and (D) for PSA 4–10 ng/ml]. On the *X*-axis, each feature’s contribution is shown. A feature with a negative Shapley value will favourably impact the prediction (decrease the risk of dying). The *Y*-axis indicated the value of the feature itself, with a high value (in red) is associated with a positive Shapley value that was associated with increased risk of HGPCa, while a low value (in blue) was associated with decreased Shapley value and the risk of HGPCa. SHAP, Shapley Additive exPlanations.

The APCA score outperformed PSA and fPSA in diagnostic accuracy in predicting PCa and HGPCa in the multi-centre and independent validation cohorts. According to the Shapely Additive Explanations (SHAP) value from the APCA score, the most dominant predictor for HGPCa patients in the APCA score was the size of the prostate measured by the two dimensions (left-right diameter*up-lower diameter, LR*UL), blood gamma-glutamyl transpeptidase, age, fPSA/PSA, followed by PSA, blood neutrophil count, urinary specific gravity, serum albumin, and lymphocyte percentage (Fig. 5). In addition to previously recognized predictors (such as PSA, fPSA, fPSA/PSA, and prostate size in two dimensions), several less recognized predictors were also included, including urinary specific gravity, serum alkaline phosphatase, albumin, and glucose. As illustrated, features such as age, PSA, SG, and glucose were positively associated with PCa risk, while features such as GGT and fPSA/PSA were negatively associated with PCa risk. The predictors for HGPCa patients in the APCA score are shown in Supplementary Figure S5 in Supplemental Digital Content 2, Supplemental Digital Content 3, http://links.lww.com/JS9/B365.

### Unnecessary biopsies avoided using the APCA score

The selection of an optimal cutoff value is essential to facilitate the application of the APCA score to maximize unnecessary biopsy reduction while maintaining an acceptable rate of missing HGPCa. By setting the APCA score cutoff values at 0.0039 and 0.0201 in the multi-centre validation cohort, unnecessary biopsies could be reduced by 20.2% and 38.4%, at the risk of missing 5.0% and 10.0% of HGPCa, respectively. The APCA score performed well in the multi-centre validation cohort in the decision curve analysis. In the clinically relevant threshold range (20.0–40.0%), the net benefit of the APCA score was significantly higher than that of the fPSA/PSA or PSA (Fig. 3). At a risk threshold of 25.0% to perform a prostate biopsy, the APCA score could reduce 652 cases (30.7%) of unnecessary biopsies while missing 49 cases (7.2%) of HGPCa. At a risk threshold of 20.0%, the APCA score could reduce unnecessary biopsies by 24.6%, while only missing 5.4% of HGPCa (or 5.8% of all PCa). For other cutoff values, the correspondence between unnecessary biopsies spared ratio and HGPCa Missed Ratio is also shown in detail. These results suggested good performance of the APCA score in the multi-centre scenario (Table 4).

**Table 4:** Number of any prostate cancer(PCa) and high-grade PCa (HGPCa) missed and reduction in biopsies according to threshold probability in the range of 10-40% for the APCA score.

Probability cutoff, %	HgPCa missed, *N* (%)	PCa missed, *N* (%)	Unnecessary biopsies spared, *N* (%)
15	30 (4.4)	31 (3.2)	389 (18.3)
20	37 (5.4)	56 (5.8)	523 (24.6)
25	49 (7.2)	76 (7.9)	652 (30.7)
30	64 (9.4)	99 (10.3)	777 (36.6)
35	83 (12.2)	131 (13.6)	849 (46.1)
40	97 (14.2)	160 (16.6)	1477 (52.2)

APCAI, Asian Prostate Cancer; HGPCa, high-grade prostate cancer; PCa, prostate cancer.

Probability cutoff: the threshold value at which the decision changes in Decision Curve Analysis (DCA), offering a balance between the benefits (true positive rate) and harms (false positive rate) of the predictive model in the context of clinical decision-making.

## Discussion

### Routine health check-up data in cancer detection

The diagnosis of PCa and HGPCa was based on prostate biopsy, which was associated with unpleasant experiences, high medical costs, and possible complications such as infections. Therefore, reducing unnecessary prostate biopsies is of great medical and social importance. Although mpmRI and novel biomarkers have gained popularity in the past decades, they are not clinically available or economically feasible for providing mpmRI or novel biomarkers for all men with elevated PSA levels, especially in Asian countries with large populations. In this context, a quick, accurate, and affordable method, with no extra cost, to screen patients at a higher risk of HGPCa may help reduce the number of unnecessary biopsies. In this study, w**e** established an AI-based prediction model for HGPCa by introducing multimodal routine health check-up data that included laboratory tests, imaging examinations, and demographic information. The APCA score was performed steadily in the multi-centre scenario and in independent international cohorts with different ethical and clinical backgrounds. Although the absolute AUC value of APCA was not very high in every cohort, the AUC increase compared with that of PSA was substantial.

Recently, there have been reports on the use of routine health check-up data to predict the risk of other cancer types with AI algorithms^6,7,14^. For instance, using standard laboratory records from a health maintenance organization, researchers have established a risk score for predicting the presence of colon cancer^15^. Erdem and colleagues analyzed the Imbra dataset, including routine blood tests from breast cancer patients and healthy controls, to build a prediction model for breast cancer diagnosis that contained nine quantitative features and one dichotomous feature^7^. Furthermore, there have only been a few preliminary reports on the prediction of PCa and other cancers using routine laboratory tests. Hood *et al.* ^16^ applied an AI-based algorithm to construct a prediction model using high-dimensional peripheral blood flow cytometric natural killer cell subset phenotyping data to predict PCa. However, these studies were mainly experimental and theoretical because of the limited number of clinical features collected.

### Comparison of the APCA score and other prediction models

In this study, we screened a series of 117 features and selected 18 features for modelling based on systematic testing using seven AI-based algorithms. Previously, there had been many PCa risk prediction models published in the past two decades. Although these models showcase higher AUC absolute values, their improvement over Prostate-Specific Antigen (PSA) is far less significant than our model. Even in a well-established comprehensive prediction model^17^, the AUC increment of the prediction model vs. PSA level was limited (usually <0.1). On the other hands, using DeLong’s test, the AUCs of APCA score were significantly higher than PSA (*P* < 0.001), with an AUC increment of 0.16 (95% CI:0.13–0.20). We suppose that it is attributed to the stronger generalization and anti-overfitting abilities exhibited by AI-based algorithms when compared to traditional algorithms. The AI-based algorithms are not a simple calculation of different testing results but a summary or learning information directly from data without relying on a predetermined equation as a model. Based on these advantages, AI-based algorithms have been used for PCA management in recent years^18^. For instance, it has been confirmed that applying machine-learning approaches to PCa risk prediction in previous models could improve prediction efficacy^19^. In addition, Jungyo *et al.* ^11^. introduced a machine-learning approach to predict PCa risk with a substantial increase in accuracy. However, despite the novel statistical approach applied, the researchers only included seven well-established predictors, such as prostate-specific antigen (PSA) and free PSA, and one novel but less available predictor (testosterone level). The strength of AI-based algorithms is that they can combine many predictors, yet including a few predictors, which diminishes the advantage of AI-based algorithms. In this study, we maximized the strength of AI-based algorithms by including all available features. APCA score facilitates easier acquisition of all features compared to other models, which not only broadens the application scope of the prostate cancer risk prediction model but also addresses the usual scenario where most male prostate cancer patients are decided for biopsy based on abnormal check-up data. Risk prediction based on check-up data could cover a broader population, providing patients with an earlier risk assessment and stratification, which is crucial for deciding treatment strategies. Using AI-based risk scoring models, we were able to conduct a risk assessment of prostate cancer for each elderly patient with slight PSA abnormalities during a health check-up.

Furthermore, the design of this study is more comprehensive and rigorous than that of other studies. Studies by Jungyo *et al.* ^11^, Perera *et al.* ^19^, Tang *et al.* ^20^, and Wu *et al.* ^21^. focused solely on internal validation; this study took this step further. Not only did the study include external validation, but the APCA score also demonstrated high predictive performance in a multi-centre external validation cohort. This study included more centres than the studies by Chen *et al.* ^22^, Suzuki *et al.* ^23^, and Yoon *et al.* ^24^, and the APCA model showed a significant improvement compared to PSA in multi-centre validation (Table 5). These made the model construction and optimization more considerate of the extraction and utilization of disease data features, rather than solely focusing on data feature extraction and utilization from specific centres or certain centres. Supplementary Figure S6, Supplemental Digital Content 3, http://links.lww.com/JS9/B365 shows the distribution of APCA scores in men with PSA 4.0–10.0 ng/ml and PSA 10.0–20.0 ng/ml by cohorts. Supplementary Figure S7, Supplemental Digital Content 3, http://links.lww.com/JS9/B365 shows the heterogeneity in the distribution of 18 features among the different cohorts. Despite the heterogeneity of the features, the APCA score demonstrated a high AUC and AUC increment compared with PSA, illustrating its high generalization ability.

**Table 5:** Comparison of the APCAI score and previous AI-based or logistic regression-based prediction models.

Study	Cases	Major population	No. centres	External validation	Methodology	Predictors	AUC of model	AUC of PSA	AUC increase	Reference
This study	5194	Asian	8	Yes	AI	18	0.76	0.60	0.16	NA
Asian-ERSPC	5220	Adapted Asian	2	Yes	LR	5	0.76	0.68	0.08	^25^
Jungyo *et al.*	3791	Asians	1	No	AI	8	0.87	NA	NA	^26^
Tang *et al.*	535	Asian	1	No	LR	4	0.848	0.797	0.051	^27^
Wu *et al.*	682	Asian	1	No	LR	6	0.849	0.827	0.022	^28^
Chen *et al.*	1835	Asian	5	Yes	LR	5	0.801	0.705	0.096	^29^
Suzuki *et al.*	834	Asian	3	Yes	LR	5	0.818	0.698	0.12	^30^
Yoon *et al.*	842	Asian	2	Yes	LR	4	0.8	0.72	0.08	^31^

AI, artificial intelligence; APCAI, Asian Prostate Cancer AI; AUC, area under the curve; LR, logistic regression; NA, not applicable; PMID, PubMed Identifier; PSA, prostate-specific antigen.

### The performance of the APCA score was associated with the percentage of missing data

**Figure 6:**
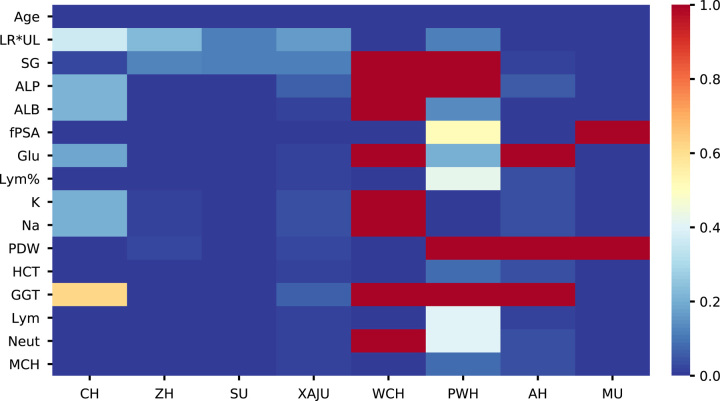
Heat map indicating missing value of different features in different centres. The *Y*-axis indicated the name of the features and the X-axis indicated different centres. The colour of each column represented the percentage of missing value in the specific feature.

The predictive performance of the APCA score varied among different cohorts. We suggest that the performance of the APCA score was associated with the percentage of missing data. As shown in Figure 6, the ZH, XAJU, and SU cohorts had the lowest percentages of missing data, and the AUCs of the three cohorts were 0.83, 0.88, and 0.77, respectively. While there was a high percentage of missing data in the WCH, PWH, and AH cohorts, the predictive performance was lower (AUC of 0.75, 0.72, and 0.72, respectively). In the MU cohort with a moderate AUC of 0.76, the percentage of missing data was moderate, but the well-known feature fPSA was not available in most cases. This may explain the lower AUC of the MU cohort in this study. In all cohorts, the APCA score achieved a substantial enhancement in AUC compared to other major predictors, such as PSA. We suggest that the APCA score could achieve high predictive performance if there were adequate predictive features; moreover, it could also achieve acceptable predictive performance in cohorts with a moderate amount of missing data. This nature of the APCA score could be an important advantage in the health check-up scenario, in which missing data may often be seen in many men.

### Interpretability of the APCA score

The black-box phenomenon is a significant challenge in the clinical application of AI-based methods. The SHAP value-based tree interpreter can improve the comprehension of model structure and interactions to improve clinical applicability. According to the local explanation using SHAP in the APCA model, age, PSA, urinary specific gravity, and glucose were positively associated with an increased HGPCa risk, whereas features such as GGT and fPSA/PSA were negatively associated with HGPCa risk. However, some of these features are not well known to be associated with PCa. However, a literature review has yielded evidence of possible associations between these features and HGPCa. For instance, albumin levels and lymphocyte counts were associated with PCa in a recent study^20^. Interestingly, a laboratory-wide association study of PCa survival using the database of the Veterans Health Administration indicated that alkaline phosphatase, albumin, haematocrit, gamma-glutamyl transpeptidase, and sodium were associated with the survival of patients with PCa^21^. Similar results were observed between lung cancer risk and routine laboratory tests^6^, as well as the risk of 18 types of cancer and C-reactive protein levels^22^. We suggest that these features are indeed associated with PCa, yet in a non-linear way, and traditional non-AI algorithms cannot detect such an association. We then corroborated the results from the SHAP by examining the correlation between different features, as well as their correlation with APCA score, using Spearman analysis. For instance, the correlation between Sodium and Albumin is reasonable because both are related to body fluid volume. Overall, there were no highly correlated variables, except for fPSA & PSA, and Sodium & Albumin (Supplementary Figure S8, Supplemental Digital Content 3, http://links.lww.com/JS9/B365).

### Clinical application of the APCA score

The application of the APCA score might be explored in the following clinical scenarios. First, it could be applied after the observation of moderately elevated PSA levels (PSA 4–20 ng/ml) in elderly men. Current standard-of-care includes prostate biopsy based on patient-physician shared decision-making or time-consuming, more expensive, and less accessible approaches (including mpMRI^23^, p2PSA, 4Kscore, and PCA3^24^, etc.). However, offering these novel tests to all patients is not practical due to limited medical resources and high costs, especially in developing Asian countries. By applying the APCA score before these expensive tests, the APCA score could be incorporated into the current diagnostic workflow. We suppose that it is attributed to the efficiency and simplicity are the strengths of the APCA score. The 18 predictors, sounds every complex, were all from basic examinations based on annual health check-ups. Most predictors were commonly applied in routine health check-ups and are very quick, affordable, and accessible, even in some remote countries or regions. Second, the APCA score could be applied to routine health check-ups. Most of the predictors of the APCA score were included in routine health check-ups of elderly males, enabling the application of the APCA score in medical check-up centres. Moreover, the APCA could be applied in patients with several missing features as well. This characteristic enhanced its advantage of simplicity and efficiency. In the future, the APCA score could be applied in a labour-free manner. The APCA system could be integrated with the health check-up systems. The APCA score could be automatically calculated and presented in the annual health check-ups report. For men without previously detected elevated PSA levels, the APCA score can detect and monitor the risk of PCa in these patients. Finally, mpMRI and novel biomarkers could be added to the APCA score to improve its strength, as previously illustrated^32^.

The application of the APCA score could reduce 20.2% and 38.4% of unnecessary biopsies at the cost of missing 5.0% and 10.0% of HGPCa cases in this round of annual health check-ups, respectively. We adjusted the probability cutoff value to meet different clinical needs in different scenarios (Table 4). To further improve the application of the APCA score, a curve plot visualizing the trade-off between missing HGPCa cases and reducing unnecessary biopsies were shown. Another curve plot illustrating the reduced unnecessary biopsies at different cutoff values (Fig. 4). The urologists can therefore discuss the risk of missing HGPCa with the patient to decide if prostate biopsy should be performed in patients with intermediate risk. For the screening of PCa before biopsy, we can use a higher probability cutoff value to reduce unnecessary biopsies. When we provide an auxiliary diagnosis, we can use a lower probability cutoff value to provide recommendations for subsequent examinations, including mpMRI and novel biomarkers.

### Economic benefit of the APCA score

**Figure 7:**
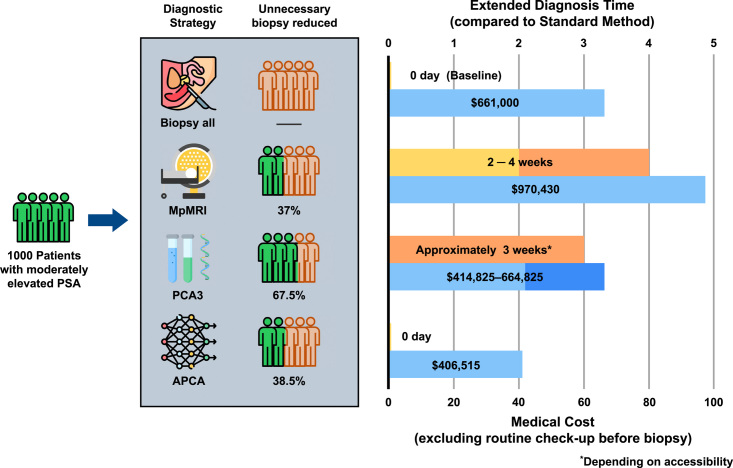
Economic benefit of the APCA score. Comparison of the medical cost and length of extended diagnostic period based on traditional strategy of biopsy all, and novel strategies based on mpMRI, PCA3 test, and the APCA score before prostate biopsy.

For example, if taking 1000 men with moderately elevated PSA and not considering the cost of the routine check-up before biopsy, the medical cost of the traditional pathological evaluation would be $661 000^33^. To compare the efficacy of the mpMRI, PCA3, and APCA score, we set the threshold of missing HGPCa at 10.0%, which means 100 patients with HGPCa might be missed if adopting the following policy: If all patients received prostate mpMRI to determine the need for prostate biopsy, 37.0% of unnecessary biopsies could be reduced^34^, at a cost of nearly $970,430 (the MR imaging cognitive procedure price was $554, data source: *www.CMS.gov*.)^33^. If all patients underwent the PCA3 test to determine the need for prostate biopsy, 67.5% unnecessary biopsies could be reduced^35^, at a cost of $414,825–664,825^36^. By introducing the APCA score, 38.4% of unnecessary biopsies could be reduced, which would reduce the cost of patients undergoing prostate biopsy (Fig. 7). In developing countries with limited medical resources, APCA may be reduce the burden on the medical service system.

This study had some limitations. First, it mainly involved Asian patients; therefore, further adaptation for Caucasian patients is warranted. Second, we introduced the dimensions of the prostate from the B ultrasound to the model, and there might be inter- and intra-rater differences among the different centres. Furthermore, Ultrasound images were also excluded. Finally, the absolute predictive accuracy was still not high. However, the increase in the AUC from PSA was relatively satisfactory.

## Conclusions

The APCA score based on routine health check-ups could reduce unnecessary prostate biopsies without additional examinations. The APCA score could help urologists to reduce the number of patients requiring prostate biopsy in Asian populations. Further prospective population-based studies are warranted to confirm the results of this study.

## Ethical approval

The protocol was approved by the Institutional Ethics Committee of Shanghai Changhai Hospital (CHEC2020-157).

## Data availability statement

Data related to our study, which includes non-human participant data and scripts, will be made available upon publication. Research partners can access these resources by making a reasonable request to the corresponding author, Dr. Rui Chen, at drchenrui@foxmail.com. It is important to note that these data and scripts are intended solely for research purposes. The availability mechanism is designed to facilitate collaboration, where data and scripts will be shared with research partners following an appropriate agreement process. This process ensures that all data-sharing adheres to the necessary ethical and legal standards. There are no additional supporting documents associated with this data sharing.

## Consent

We obtained approval from the ethics committee. Due to the retrospective design of this study, the requirement for informed consent was waived by the ethics committee. We have taken great care to respect the privacy of our patients and volunteers. The manuscript does not contain any names, initials, or hospital numbers of patients. We've made every effort to omit any identifying details that aren't essential to the scientific understanding of the study. All identifying details are omitted and they will not distort scientific meaning.

## Source of funding

This study is supported by the National Natural Science Foundation of China (82272905), the Rising-Star Program of the Science and Technology Commission of Shanghai Municipality (21QA1411500), and the Shanghai Action Plan for Technological Innovation Grant (No. 22ZR1478000, 22ZR1415300, 22511104000, 23S41900500).

## Author contribution

Z.S., W.Z.: conceptualization, methodology, data curation, project administration, resources, writing—original draft, writing—review and& editing. Q.J., L.D., L.D.: visualization, methodology, formal analysis, project administration, investigation, writing—original draft. W.M., Y.L., W.Z., Y.Y., J.L., K.L.: formal analysis, writing—review and editing. J.Y.P., C.-F.N., O.T.A., Q.W., L.L., X.W., M.C.: resources, data curation, investigation, project administration. Z.C., F.W., R.C.: conceptualization, formal analysis, data curation, investigation, methodology, project administration, supervision, validation, writing—review and editing.

## Conflicts of interest disclosure

The authors declare no conflicts of interest.

## Research registration unique identifying number (UIN)

The protocol was registered on the Chinese Clinical Trial Registry (http://www.chictr.org.cn, ChiCTR2100048428).

## Guarantor

Rui Chen and Zhixing Cao serve as the Guarantors for this work. They accept full responsibility for the entirety of the work, the conduct of the study, had access to all the data, and controlled the decision to publish.

## Provenance and peer review

Not invited.

## Supplementary Material

SUPPLEMENTARY MATERIAL
