# Supplementary material for: Artificial intelligence-aided detection for prostate cancer with multimodal routine health check-up data: an Asian multi-center study
Source: Int J Surg. 2023 Nov 20;109(12):3848–60. doi: 10.1097/JS9.0000000000000862 (PMC10720852; doi:10.1097/JS9.0000000000000862)
Supplement: SUPPLEMENTARY MATERIAL [file js9-109-3848-s003.docx]

SUPPLEMENTARY MATERIAL

**Artificial intelligence-aided detection for prostate cancer with multi-modal routine health check-up data: an Asian multi-center study**

[Supplement Table 1. All features under consideration in the prediction model 2](#_Toc28888)

[Supplement Table 2. Features included in the prediction model 7](#_Toc24825)

[Supplement Table 3. AUCs of receiver operator characteristic curves, sensitivity, specificity, positive predictive value, and negative predictive value of the APCA score when predicting PCa. 8](#_Toc26262)

[Supplement Table 4. AUCs of receiver operator characteristic curves for prediction model and individual predictors when predicting PCa at PSA 4-20ng/ml. 9](#_Toc10736)

[Supplement Figure 1. ROC curves for the APCA score in predicting HGPCa, PSA, fPSA/PSA in internal validation cohorts and seven external validation cohorts, with different PSA ranges for different panels 10](#_Toc8383)

[Supplement Figure 2. Decision Curve Analysis for the APCA score in predicting HGPCa, PSA, fPSA/PSA in an internal validation cohort and seven external validation cohorts 11](#_Toc360)

[Supplement Figure 3. ROC curves, calibration plot, and decision curve analysis of the APCA Score in predicting PCa in men with PSA of 4.0-10.0ng/ml and PSA of 4.0-20.0ng/ml. 12](#_Toc14621)

[Supplement Figure 4. Waterfall plot of the APCA score related to PCa patients in the multi-center validation cohort with (A) PSA 4.0-20.0ng/ml and (B) 4.0-10.0ng/ml. 13](#_Toc18974)

[Supplement Figure 5. The impact of different features illustrated by SHAP value in PCa prediction. 14](#_Toc24360)

[Supplementary Figure 7. Stacked violin plot of feature distribution across each cohort. 16](#_Toc13976)

[Supplementary Figure 8. Correlation matrix among different features and among different features and the APCA scores. 17](#_Toc24723)

# Supplement Table 1. All features under consideration in the prediction model

| **Number** | **Features-qualitative/quantitative-Unit-Reference Range** | **Sample Type** | **Grouping of Features** |
| --- | --- | --- | --- |
| 1 | Age-Quantitative | Demographic | Demographic |
| 2 | BMI | Demographic | Demographic |
| 3 | Left and right diameter _ upper and lower diameter (cm)-quantitative [ [*]] | B-ultrasound | B-ultrasound |
| 4 | Blood _ Prostate Specific Antigen (PSA) -Quantitative [ [ng/ml *]] | Blood | Prostate Specific Antigen |
| 5 | Blood _ Free PSA (F-PSA)-Quantitative [ [ng/ml *]] | Blood | Prostate Specific Antigen |
| 6 | Blood _ FPSA/PSA-Quantitative [ [*]] | Blood | Prostate Specific Antigen |
| 7 | Blood α-hydroxybutyrate dehydrogenase-qualitative | Blood | Blood Biochemistry |
| 8 | Blood γ-glutamyl transpeptidase-qualitative | Blood | Blood Biochemistry |
| 9 | Serum Alanine Aminotransferase (AlT) -qualitative | Blood | Blood Biochemistry |
| 10 | Blood _ Alanine Aminotransferase (AlT) -Quantitation [ [u/l * [9.0,50.0]]] | Blood | Blood Biochemistry |
| 11 | Blood _ Lactate Dehydrogenase-Qualitative | Blood | Blood Biochemistry |
| 12 | Blood _ Low Density Lipoprotein Cholesterol-Qualitative | Blood | Blood Biochemistry |
| 13 | Blood _ Prealbumin-Qualitative | Blood | Blood Biochemistry |
| 14 | Blood urea-qualitative | Blood | Blood Biochemistry |
| 15 | Blood _ uric acid-qualitative | Blood | Blood Biochemistry |
| 16 | Blood _ Total Cholesterol-Qualitative | Blood | Blood Biochemistry |
| 17 | Blood _ Total Bilirubin (T-BIl)-Qualitative | Blood | Blood Biochemistry |
| 18 | Blood _ Total Protein-Qualitative | Blood | Blood Biochemistry |
| 19 | Blood _ Total Protein-Quantitative [ [G/l * [65.0, 85.0]]] | Blood | Blood Biochemistry |
| 20 | Blood _ Chloride Ion (Cl)-Qualitative | Blood | Blood Biochemistry |
| 21 | Blood _ Chloride Ion (Cl)-Quantitative [ [mmol/l * [99.0, 110.0]]] | Blood | Blood Biochemistry |
| 22 | Blood _ Globulin-Qualitative | Blood | Blood Biochemistry |
| 23 | Blood _ Globulin-Quantitative [ [G/l * [20.0, 40.0]]] | Blood | Blood Biochemistry |
| 24 | Blood _ Triglycerides-Qualitative | Blood | Blood Biochemistry |
| 25 | Blood _ Albumin-Qualitative | Blood | Blood Biochemistry |
| 26 | Blood _ Albumin-Quantitative [ [G/l * [40.0, 55.0]]] | Blood | Blood Biochemistry |
| 27 | Blood _ Albumin/Globulin-Qualitative | Blood | Blood Biochemistry |
| 28 | Blood _ Albumin/Globulin-Quantitative [ [* [1.2,2.4]]] | Blood | Blood Biochemistry |
| 29 | Blood _ Direct Bilirubin (D-BIl)-Qualitative | Blood | Blood Biochemistry |
| 30 | Blood Alkaline Phosphatase-Qualitative | Blood | Blood Biochemistry |
| 31 | Blood _ Alkaline Phosphatase-Quantitative [ [u/l * [45.0,125.0]]] | Blood | Blood Biochemistry |
| 32 | Blood _ Phosphorus Ion (P)-Qualitative | Blood | Blood Biochemistry |
| 33 | Blood _ Fibrinogen-Qualitative | Blood | Blood Biochemistry |
| 34 | Blood _ Creatinine-Qualitative | Blood | Blood Biochemistry |
| 35 | Blood _ Creatine Kinase (CK)-Qualitative | Blood | Blood Biochemistry |
| 36 | Blood _ Plaquetocrito-Qualitative | Blood | Blood Biochemistry |
| 37 | Blood _ Lipase-Quantitative [ [u/l * [1.0, 60.0]]] | Blood | Blood Biochemistry |
| 38 | Blood _ Glucose (Glu)-Qualitative | Blood | Blood Biochemistry |
| 39 | Blood _ Glucose (Glu)-Quantitative [ [mmol/l * [3.9,6.1]]] | Blood | Blood Biochemistry |
| 40 | Blood _ hypersensitive C-reactive protein-Qualitative（hs-CRP） | Blood | Blood Biochemistry |
| 41 | Blood _ hs-CRP-Quantitative [ [mg/l * [0.0, 3.0]]] | Blood | Blood Biochemistry |
| 42 | Blood _ Calcium (Ca)-Qualitative | Blood | Blood Biochemistry |
| 43 | Blood _ Sodium (0 a)-Qualitative | Blood | Blood Biochemistry |
| 44 | Blood _ Sodium (0 a)-Quantitative [ [mmol/l * [137.0,147.0]]] | Blood | Blood Biochemistry |
| 45 | Blood _ Potassium Ion (K)-Qualitative | Blood | Blood Biochemistry |
| 46 | Blood _ Potassium Ion (K)-Quantitative [ [mmol/l * [3.5,5.3]]] | Blood | Blood Biochemistry |
| 47 | Blood _ Unconjugated Bilirubin-Qualitative | Blood | Blood Biochemistry |
| 48 | Blood _ High Density Lipoprotein Cholesterol-Qualitative | Blood | Blood Biochemistry |
| 49 | Blood _ Gamma-Glutamyl Transpeptidase-Quantitation [ [u/l * [10.0, 60.0]]] | Blood | Blood Routine Test |
| 50 | Blood _ Neutrophil Percentage (NEUT%)-Qualitative | Blood | Blood Routine Test |
| 51 | Blood _ Neutrophil Percentage (NEUT%)-Quantitative [ [* [0.4,0.75]]] | Blood | Blood Routine Test |
| 52 | Blood _ Neutrophil Absolute Count (NEUT #)-Qualitative | Blood | Blood Routine Test |
| 53 | Blood _ Neutrophil Absolute Count (NEUT #)-Quantitative [ [10 ^ 9/l * [1.8,6.3]]] | Blood | Blood Routine Test |
| 54 | Blood _ Percentage of Monocytes-Qualitative | Blood | Blood Routine Test |
| 55 | Blood _% Monocytes-Quantitative [ [* [0.03,0.1]]] | Blood | Blood Routine Test |
| 56 | Blood _ Absolute Monocyte Count-Qualitative | Blood | Blood Routine Test |
| 57 | Blood _ Absolute Monocyte Count-Quantitative [ [10 ^ 9/l * [0.1, 0.6]]] | Blood | Blood Routine Test |
| 58 | Blood _ Basophil Percentage (BASO%)-Qualitative | Blood | Blood Routine Test |
| 59 | Blood _ Basophil Percentage (BASO%)-Quantitative [ [* [0.0, 0.01]]] | Blood | Blood Routine Test |
| 60 | Blood _ Basophil Absolute Count (BASO #)-Qualitative | Blood | Blood Routine Test |
| 61 | Blood _ Basophil Absolute Count (BASO #)-Quantitative [ [10 ^ 9/l * [0.0, 0.06]]] | Blood | Blood Routine Test |
| 62 | Blood _ Eosinophil Percentage (EO%)-Qualitative | Blood | Blood Routine Test |
| 63 | Blood _ Eosinophil Percentage (EO%)-Quantitative [ [* [0.004,0.08]]] | Blood | Blood Routine Test |
| 64 | Blood _ Absolute Eosinophil Count (EO #)-Qualitative | Blood | Blood Routine Test |
| 65 | Blood _ Absolute Eosinophil Count (EO #)-Quantitative [ [10 ^ 9/l * [0.02,0.52]]] | Blood | Blood Routine Test |
| 66 | Blood _% Large Unstained Cells-Qualitative | Blood | Blood Routine Test |
| 67 | Blood _% Large Unstained Cells-Quantitative [ [* [0.0, 0.04]]] | Blood | Blood Routine Test |
| 68 | Blood _ Large Unstained Cell Count-Qualitative | Blood | Blood Routine Test |
| 69 | Blood _ Large Unstained Cell Count-Quantitative [ [10 ^ 9/l * [0.0, 0.4]]] | Blood | Blood Routine Test |
| 70 | Blood aspartate aminotransferase-qualitative | Blood | Blood Routine Test |
| 71 | Blood _ Aspartate Aminotransferase-Quantitative [ [u/l * [15.0, 40.0]]] | Blood | Blood Routine Test |
| 72 | Blood _ Mean Corpuscular Volume (MCV)-Qualitative | Blood | Blood Routine Test |
| 73 | Blood _ Mean Corpuscular Volume (MCV)-Quantitative [ [fl * [82.0, 100.0]]] | Blood | Blood Routine Test |
| 74 | Blood _ Mean Corpuscular Hemoglobin (MCH)-Qualitative | Blood | Blood Routine Test |
| 75 | Blood _ Mean Corpuscular Hemoglobin (MCH)-Quantitative [ [PG * [27.0,34.0]]] | Blood | Blood Routine Test |
| 76 | Blood _ Mean corpuscular hemoglobin concentration (MCHC)-Qualitative | Blood | Blood Routine Test |
| 77 | Blood _ Mean Corpuscular Hemoglobin Concentration (MCHC)-Quantitative [ [G/l * [316.0,354.0]]] | Blood | Blood Routine Test |
| 78 | Blood _ Mean Platelet Volume (MPV)-Qualitative | Blood | Blood Routine Test |
| 79 | Blood _ Mean Platelet Volume (MPV)-Quantitative [ [fl * [6.0, 14.0]]] | Blood | Blood Routine Test |
| 80 | Blood _ Lymphocyte Percentage-Qualitative | Blood | Blood Routine Test |
| 81 | Blood _ Lymphocyte Percent-Quantitative [ [* [0.2,0.5]]] | Blood | Blood Routine Test |
| 82 | Blood _ Lymphocyte Count-Qualitative | Blood | Blood Routine Test |
| 83 | Blood _ Lymphocyte Count-Quantitative [ [10 ^ 9/l * [1.1,3.2]]] | Blood | Blood Routine Test |
| 84 | Blood _ White Blood Cell Count-Qualitative | Blood | Blood Routine Test |
| 85 | Blood _ White Cell Count-Quantitative [ [10 ^ 9/l * [3.5,9.5]]] | Blood | Blood Routine Test |
| 86 | Blood _ Red Blood Cell Distribution Width (RDW)-Qualitative | Blood | Blood Routine Test |
| 87 | Blood _ Red Cell Distribution Width (RDW)-Quantitative [ [% * [10.6, 15.0]]] | Blood | Blood Routine Test |
| 88 | Blood _ Hematocrit (HCT)-Qualitative | Blood | Blood Routine Test |
| 89 | Blood _ Hematocrit (HCT)-Quantitative [ [l/l * [0.4,0.5]]] | Blood | Blood Routine Test |
| 90 | Blood _ Red Blood Cell Count-Qualitative | Blood | Blood Routine Test |
| 91 | Blood _ Red Blood Cell Count-Quantitative [ [10 ^ 12/l * [4.3,5.8]]] | Blood | Blood Routine Test |
| 92 | Blood _ Platelet Distribution Width (PDW)-Qualitative | Blood | Blood Routine Test |
| 93 | Blood _ Platelet Distribution Width (PDW)-Quantitative [ [% * [8.7, 18.1]]] | Blood | Blood Routine Test |
| 94 | Blood _ Plaquetocrito (PCT)-Qualitative | Blood | Blood Routine Test |
| 95 | Blood _ Plaquetocrito (PCT)-Quantitative [ [l/l * [0.11, 0.28]]] | Blood | Blood Routine Test |
| 96 | Blood _ Platelet Count (PlT)-Qualitative | Blood | Blood Routine Test |
| 97 | Blood _ Platelet Count (PlT)-Quantitative [ [10 ^ 9/l * [125.0,350.0]]] | Blood | Blood Routine Test |
| 98 | Blood _ Hemoglobin (hb)-Qualitative | Blood | Blood Routine Test |
| 99 | Blood _ Hemoglobin (hb)-Quantitative [ [G/l * [130.0, 175.0]]] | Blood | Blood Routine Test |
| 100 | Stool _ Stool Red Blood Cells-Qualitative | Stool | Stool Routine Test |
| 101 | Stool _ Fecal Red Blood Cells-Quantitation [ [PCs/HP * [0.0, 0.0]]] | Stool | Stool Routine Test |
| 102 | Urine _ Other Crystals-Qualitative | Urine | Urine Routine Test |
| 103 | Urine _ Urine-Bacteria-Qualitative | Urine | Urine Routine Test |
| 104 | Urine _ Urine Specific Gravity-Qualitative | Urine | Urine Routine Test |
| 105 | Urine _ Urine Specific Gravity-Quantitative [ [* [1.003, 1.03]]] | Urine | Urine Routine Test |
| 106 | Urine _ Urine White Blood Cell-Qualitative | Urine | Urine Routine Test |
| 107 | Urine _ Urine Bilirubin-Qualitative | Urine | Urine Routine Test |
| 108 | Urine _ Urine Glucose-Qualitative | Urine | Urine Routine Test |
| 109 | Urine _ Proteinuria-Qualitative | Urine | Urine Routine Test |
| 110 | Urine _ Urine Clear Cast-Qualitative | Urine | Urine Routine Test |
| 111 | Urine _ Urine Ketone Bodies-Qualitative | Urine | Urine Routine Test |
| 112 | Urine _ uric acid alkalinity-qualitative | Urine | Urine Routine Test |
| 113 | Urine _ Urine Occult Blood-Qualitative | Urine | Urine Routine Test |
| 114 | Urine _ Urine squamous epithelial cells-qualitative | Urine | Urine Routine Test |
| 115 | Urine _ Leukocyte Esterase-Qualitative | Urine | Urine Routine Test |
| 116 | Urine _ Red Blood Cell Count-Qualitative | Urine | Urine Routine Test |
| 117 | Urine _ Nitrite-Qualitative | Urine | Urine Routine Test |

# Supplement Table 2. Features included in the prediction model

| **Features (unit)** | **Reference Range** | **Abbreviation** |
| --- | --- | --- |
| Age (yr) | NA | Age |
| Ultrasound left and right diameter * upper and lower diameter (cm2) | NA | LR*UL |
| Urinary specific gravity (%) | 1.003,1.03 | SG |
| Serum alkaline phosphatase (u/l) | 40.0,150.0 | ALP |
| fPSA/PSA | NA | fPSA/PSA |
| Serum albumin (g/l) | 35.0,50.0 | ALB |
| fPSA (ng/ml) | NA | fPSA |
| PSA (ng/ml) | 0.0,4.0 | PSA |
| Blood glucose (mmol/l) | 3.6,6.1 | Glu |
| Lymphocyte percentage (%) | 20.0,40.0 | Lym% |
| Serum potassium (mmol/l) | 134.0,145.0 | K |
| Serum sodium (mmol/l) | 3.5,4.5 | Na |
| Platelet distribution width (fl) | 9.8,16.1 | PDW |
| Blood hematocrit (%) | 32.0,52.0 | HCT |
| Gamma-Glutamyl transpeptidase (u/l) | 0.0,47.0 | GGT |
| Blood lymphocyte count (%) | 0.8,4.0 | Lym |
| Blood neutrophil count (10^9/l) | 2.0,7.0 | Neut |
| Blood mean corpuscular hemoglobin (pg) | 26.0,32.0 | MCH |

# Supplement Table 3. AUCs of receiver operator characteristic curves, sensitivity, specificity, positive predictive value, and negative predictive value of the APCA score when predicting PCa.

|  | | **CH Validation** | **Multicenter**  **validation cohort** | **ZH** | **SU** | **WCH** | **XAJU** | **PWH** | **AH** | **MU** |
| --- | --- | --- | --- | --- | --- | --- | --- | --- | --- | --- |
| PSA 4.0-10.0ng/ml | AUC | 0.98 | 0.75 | 0.83 | 0.8 | 0.71 | 0.81 | 0.67 | 0.72 | 0.73 |
|  | Sensitivity | 90.15% | 90.15% | 91.18% | 90.70% | 91.18% | 89.29% | 90.35% | 90.32% | 90.91% |
|  | Specificity | 95.31% | 33.13% | 50.00% | 42.36% | 31.71% | 40.37% | 17.71% | 31.58% | 36.78% |
|  | PPV | 90.84% | 38.28% | 29.11% | 40.00% | 35.63% | 27.78% | 56.59% | 45.31% | 35.29% |
|  | NPV | 94.94% | 87.97% | 96.18% | 91.49% | 89.66% | 93.62% | 60.71% | 83.87% | 91.43% |
| PSA 4.0-20.0ng/ml | AUC | 0.98 | 0.79 | 0.86 | 0.81 | 0.75 | 0.86 | 0.7 | 0.73 | 0.76 |
|  | Sensitivity | 90.11% | 90.05% | 90.38% | 90.00% | 90.41% | 90.00% | 90.45% | 90.38% | 91.53% |
|  | Specificity | 96.07% | 39.27% | 58.28% | 44.10% | 27.54% | 60.08% | 19.08% | 32.90% | 39.68% |
|  | PPV | 93.68% | 43.73% | 41.47% | 46.22% | 35.29% | 38.41% | 56.69% | 50.94% | 41.54% |
|  | NPV | 93.76% | 88.28% | 94.88% | 89.20% | 86.79% | 95.60% | 63.04% | 81.60% | 90.91% |

AUC, Area Under the Curve; APCAI, Asian Prostate Cancer AI; PCa, Prostate Cancer; PPV, Positive Predictive Value; NPV, Negative Predictive ValueHospital of The Chinese University of Hong Kong; UM, University of Malaya Medical Centre.

Supplement Table 4. AUCs of receiver operator characteristic curves for prediction model and individual predictors when predicting PCa at PSA 4-20ng/ml.

| **Predictors** | **CH validation** | **ZH** | **SU** | **WCH** | **XAJU** | **PWH** | **AH** | **MU** |
| --- | --- | --- | --- | --- | --- | --- | --- | --- |
| APCA Score | 0.98(0.98-0.99) | 0.86(0.83-0.90) | 0.81(0.77-0.85) | 0.75(0.68-0.82) | 0.86(0.80-0.91) | 0.70(0.64-0.75) | 0.73(0.68-0.77) | 0.76(0.68-0.84) |
| LR*UL | 0.78(0.74-0.82) | 0.77(0.73-0.82) | 0.72(0.67-0.76) | 0.70(0.63-0.77) | 0.85(0.79-0.90) | 0.71(0.65-0.76) | 0.71(0.67-0.76) | 0.71(0.62-0.79) |
| SG | 0.57(0.52-0.61) | 0.50(0.45-0.55) | 0.51(0.46-0.56) | NA | 0.55(0.47-0.62) | NA | 0.49(0.44-0.54) | 0.56(0.48-0.65) |
| ALP | 0.51(0.46-0.56) | 0.53(0.48-0.58) | 0.53(0.48-0.58) | NA | 0.50(0.42-0.58) | NA | 0.54(0.50-0.59) | 0.50(0.41-0.59) |
| fPSA/PSA | 0.65(0.61-0.70) | 0.69(0.64-0.74) | 0.65(0.60-0.70) | 0.66(0.59-0.73) | 0.64(0.56-0.72) | 0.44(0.38-0.50) | 0.61(0.56-0.65) | NA |
| ALB | 0.50(0.45-0.55) | 0.50(0.45-0.56) | 0.56(0.51-0.61) | NA | 0.53(0.45-0.61) | 0.47(0.41-0.54) | 0.49(0.45-0.54) | 0.52(0.43-0.61) |
| fPSA | 0.57(0.53-0.61) | 0.55(0.49-0.60) | 0.58(0.53-0.63) | 0.60(0.52-0.67) | 0.61(0.53-0.68) | 0.43(0.37-0.49) | 0.50(0.45-0.55) | NA |
| PSA | 0.59(0.55-0.63) | 0.64(0.58-0.69) | 0.57(0.52-0.62) | 0.52(0.44-0.60) | 0.51(0.43-0.59) | 0.52(0.46-0.59) | 0.61(0.56-0.66) | 0.61(0.52-0.69) |
| Glu | 0.51(0.47-0.56) | 0.51(0.46-0.56) | 0.56(0.51-0.61) | NA | 0.58(0.50-0.66) | 0.50(0.43-0.56) | NA | 0.50(0.41-0.58) |
| Lym% | 0.51(0.46-0.55) | 0.52(0.47-0.57) | 0.52(0.47-0.57) | 0.53(0.45-0.61) | 0.57(0.50-0.65) | 0.52(0.46-0.58) | 0.51(0.46-0.56) | 0.57(0.48-0.65) |
| K | 0.55(0.50-0.60) | 0.53(0.48-0.58) | 0.49(0.44-0.54) | NA | 0.55(0.48-0.63) | 0.52(0.45-0.58) | 0.51(0.46-0.56) | 0.50(0.41-0.59) |
| Na | 0.54(0.49-0.59) | 0.51(0.46-0.56) | 0.54(0.49-0.59) | NA | 0.51(0.43-0.59) | 0.51(0.45-0.57) | 0.47(0.42-0.52) | 0.58(0.49-0.66) |
| PDW | 0.51(0.47-0.56) | 0.49(0.44-0.54) | 0.54(0.49-0.58) | 0.50(0.42-0.59) | 0.54(0.46-0.62) | NA | NA | NA |
| HCT | 0.52(0.48-0.57) | 0.49(0.44-0.54) | 0.54(0.49-0.59) | 0.54(0.46-0.62) | 0.50(0.42-0.58) | 0.49(0.43-0.56) | 0.50(0.46-0.55) | 0.60(0.51-0.69) |
| GGT | 0.66(0.61-0.71) | 0.52(0.47-0.57) | 0.54(0.49-0.59) | NA | 0.55(0.48-0.62) | NA | NA | 0.52(0.43-0.62) |
| Lym | 0.49(0.44-0.54) | 0.47(0.42-0.53) | 0.53(0.48-0.58) | 0.53(0.45-0.61) | 0.59(0.52-0.67) | 0.53(0.47-0.59) | 0.52(0.47-0.57) | 0.49(0.40-0.58) |
| Neut | 0.51(0.46-0.55) | 0.49(0.44-0.54) | 0.51(0.46-0.57) | NA | 0.53(0.45-0.60) | 0.45(0.39-0.51) | 0.52(0.47-0.57) | 0.45(0.36-0.53) |
| MCH | 0.53(0.49-0.58) | 0.51(0.45-0.56) | 0.51(0.46-0.56) | 0.57(0.49-0.65) | 0.48(0.41-0.56) | 0.48(0.42-0.54) | 0.54(0.49-0.59) | 0.54(0.45-0.63) |

AUC, Area Under the Curve; PCa, Prostate Cancer; APCAI, Asian Prostate Cancer AI; LR*UL, Ultrasound left and right diameter * upper and lower diameter; SG, Urinary specific gravity; ALP, Serum alkaline phosphatase; fPSA/PSA, fPSA/PSA; ALB, Serum albumin; fPSA, Free Prostate-Specific Antigen; PSA, Prostate-Specific Antigen; Glu, Blood glucose; Lym%, Lymphocyte percentage; K, Serum potassium; Na, Serum sodium; PDW, Platelet distribution width; HCT, Blood hematocrit; GGT, Gamma-Glutamyl transpeptidase; Lym, Blood lymphocyte count; Neut, Blood neutrophil count; MCH, Blood mean corpuscular hemoglobin;

# Supplement Figure 1. ROC curves for the APCA score in predicting HGPCa, PSA, fPSA/PSA in internal validation cohorts and seven external validation cohorts, with different PSA ranges for different panels


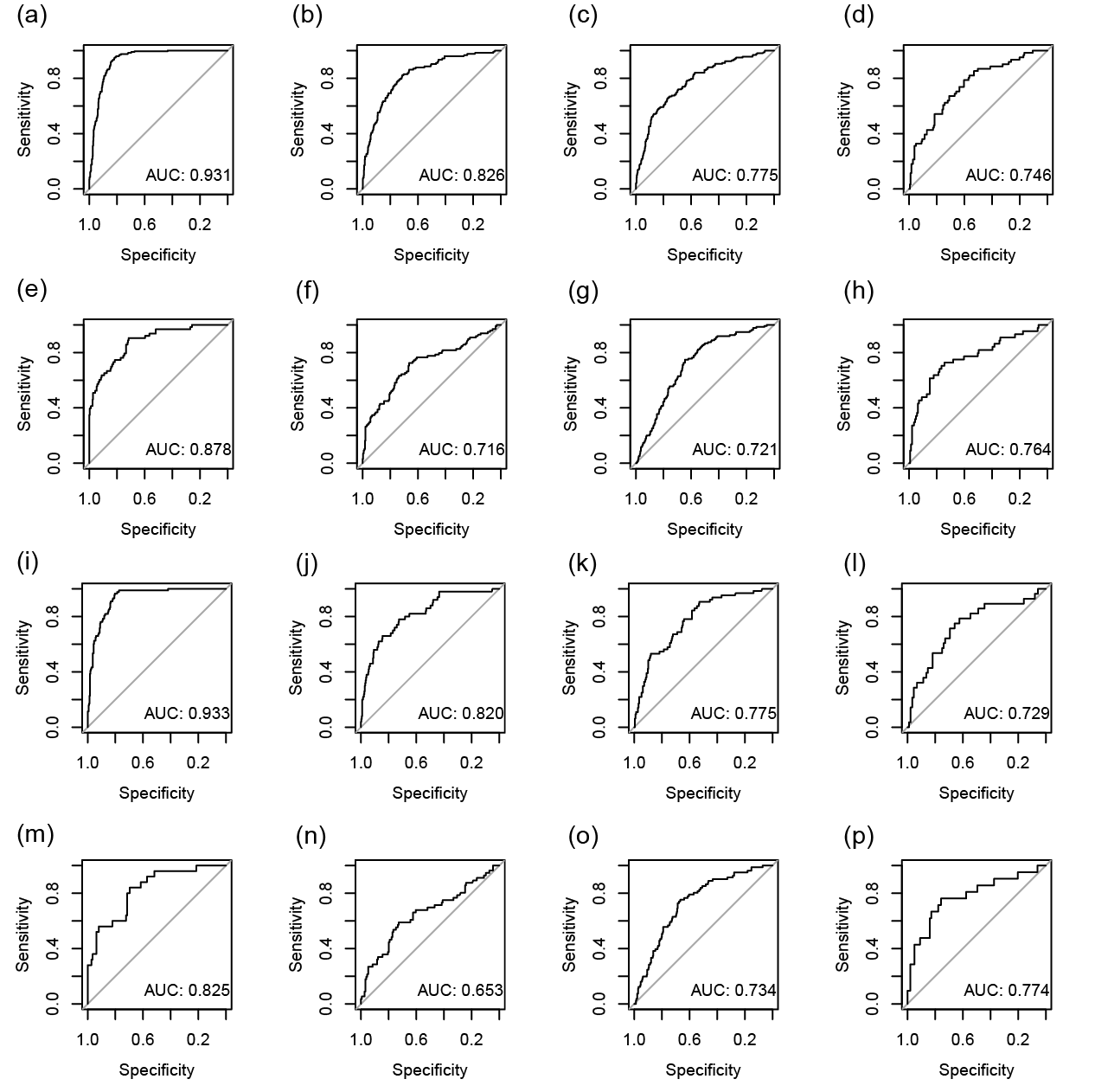


Panels a-h pertain to a PSA range of 4.0-20.0ng/ml, while panels i-p are concerned with a PSA range of 4.0-10.0ng/ml. The validation cohorts include the following hospitals: (a, i. CH. b, j. ZH. c, k. SU. d, l. WCH. e, m. XAJU. f, n. AH. g,o. PWH. h, p. UM).

# Supplement Figure 2. Decision Curve Analysis for the APCA score in predicting HGPCa, PSA, fPSA/PSA in an internal validation cohort and seven external validation cohorts


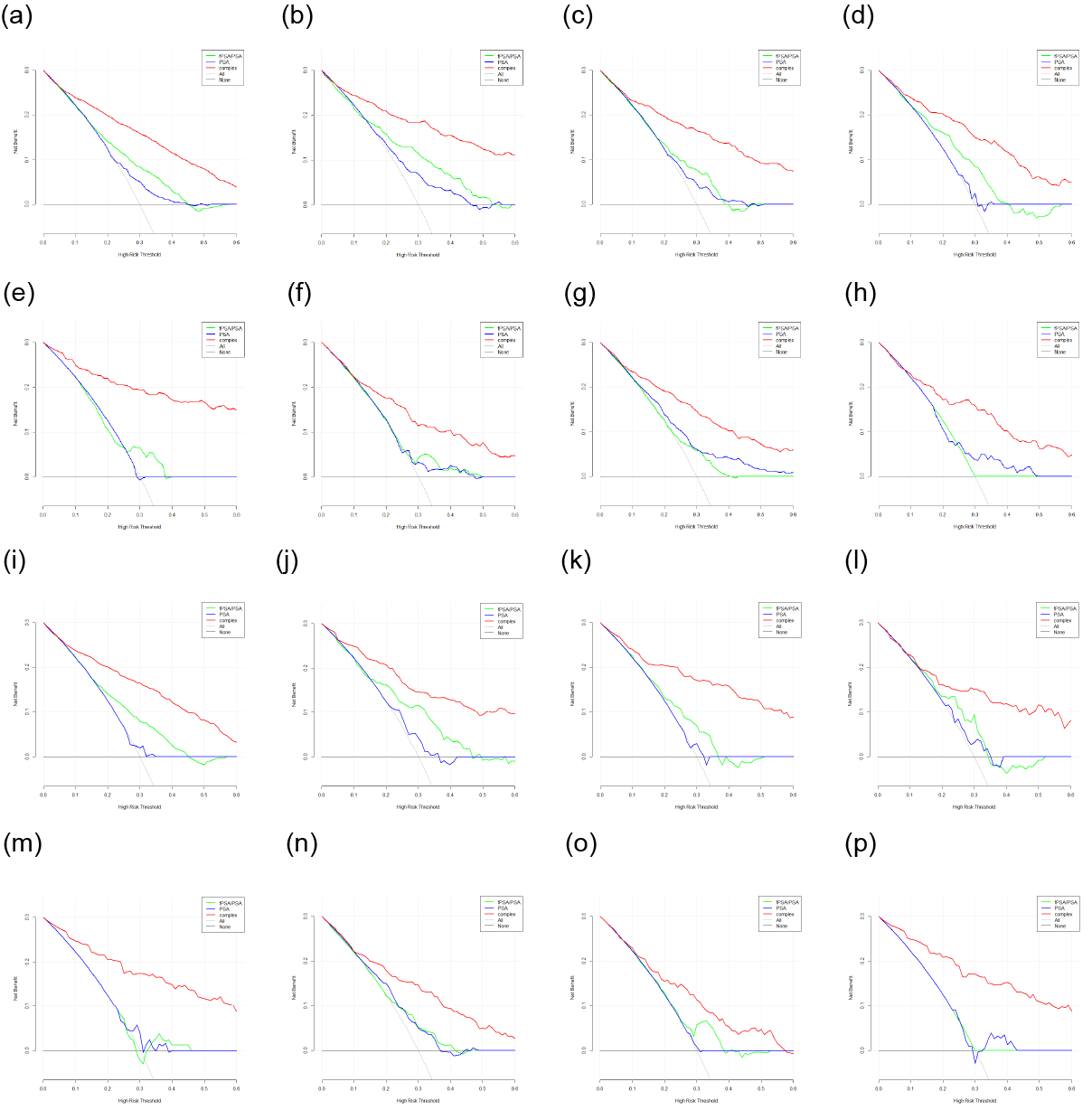


This figure provides information for both PSA ranges, 4.0-20.0ng/ml and 4.0-10.0 ng/ml. Complex means APCA score. The DCA shows that the APCA score presented the highest net benefit across all threshold probabilities for HGPCa. The horizontal black lines parallel to the x-axis represent the scenario of no patient undergoing a biopsy (Treat None), and the grey line indicates the hypothesis that all the patients will have HGPCa (Treat All). The validation cohorts include the following hospitals: (a, i. CH. b, j. ZH. c, k. SU. d, l. WCH. e, m. XAJU. f, n. AH. g,o. PWH. h, p. UM).

# Supplement Figure 3. ROC curves, calibration plot, and decision curve analysis of the APCA Score in predicting PCa in men with PSA of 4.0-10.0ng/ml and PSA of 4.0-20.0ng/ml.


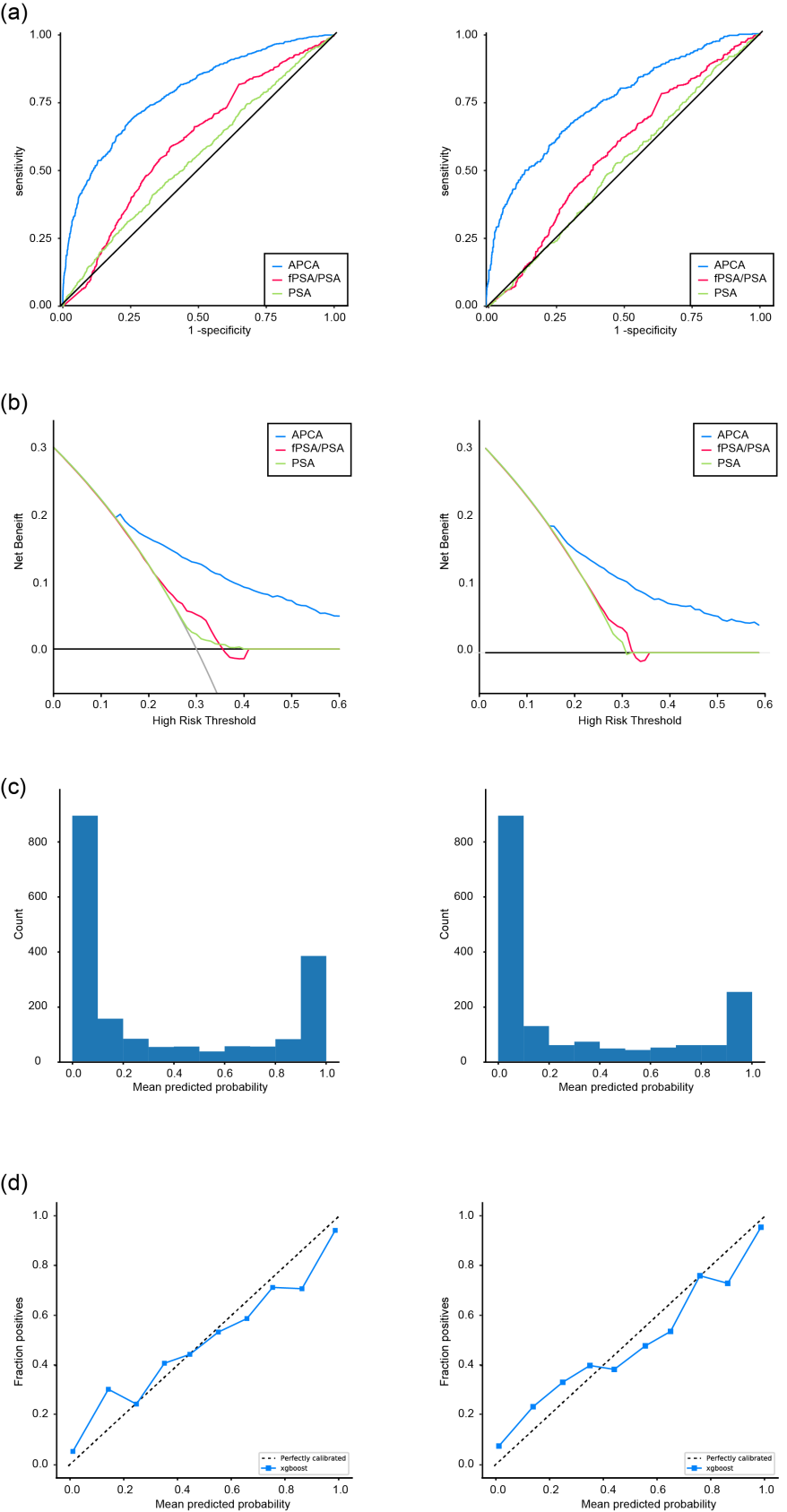


1. ROC curves; b. Calibration plot; c. Histogram of mean predicted possibility; d. Decision curve analysis with PSA 4.0-10.0ng/ml represented on the left panels and PSA 4.0-10.0ng/ml on the right panels.

# Supplement Figure 4. Waterfall plot of the APCA score related to PCa patients in the multi-center validation cohort with (A) PSA 4.0-20.0ng/ml and (B) 4.0-10.0ng/ml.


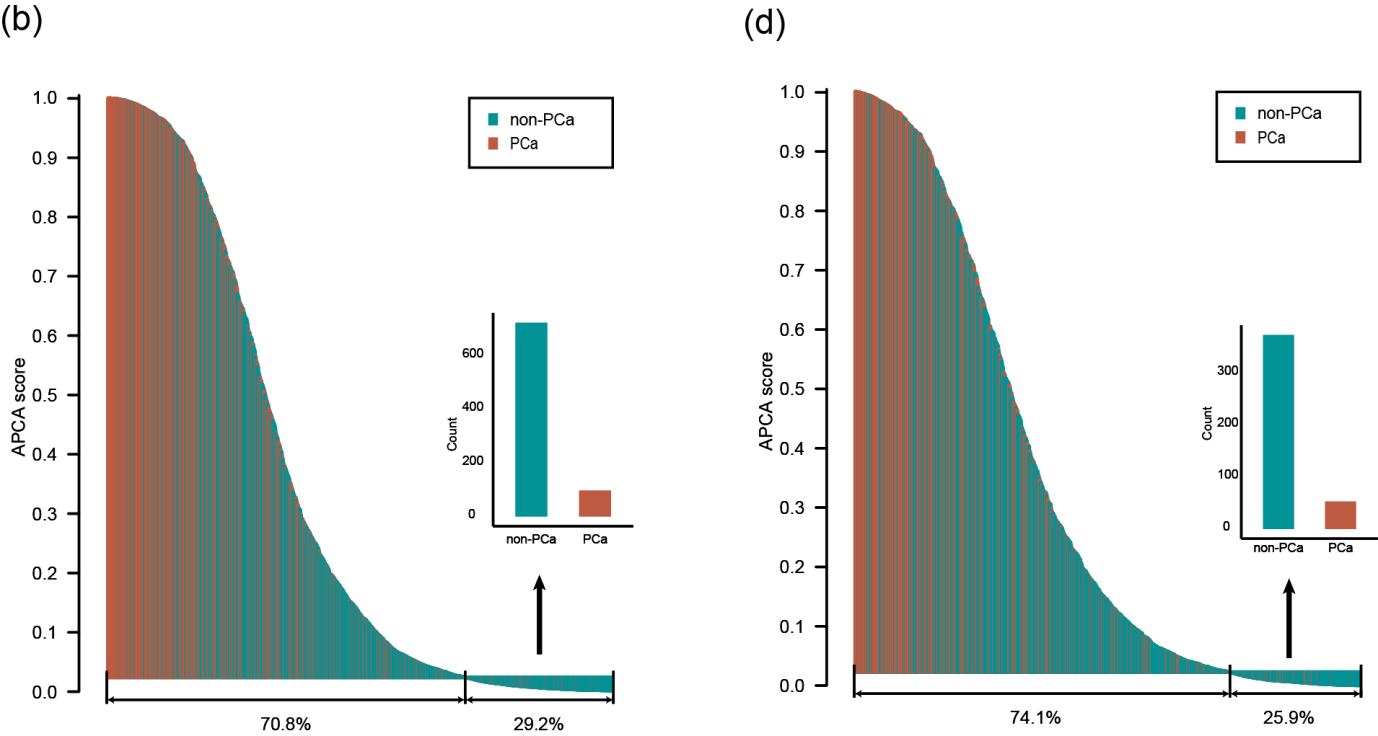


Each bar represents an individual. Red indicates PCa; blue indicates non-PCa. The horizontal lines represent the cutoff points of 0.02226 and 0.02215 at a sensitivity of 90% in men with PSA 4.0-20.0ng/ml and 4.0-10.0ng/ml, respectively.

# Supplement Figure 5. The impact of different features illustrated by SHAP value in PCa prediction.


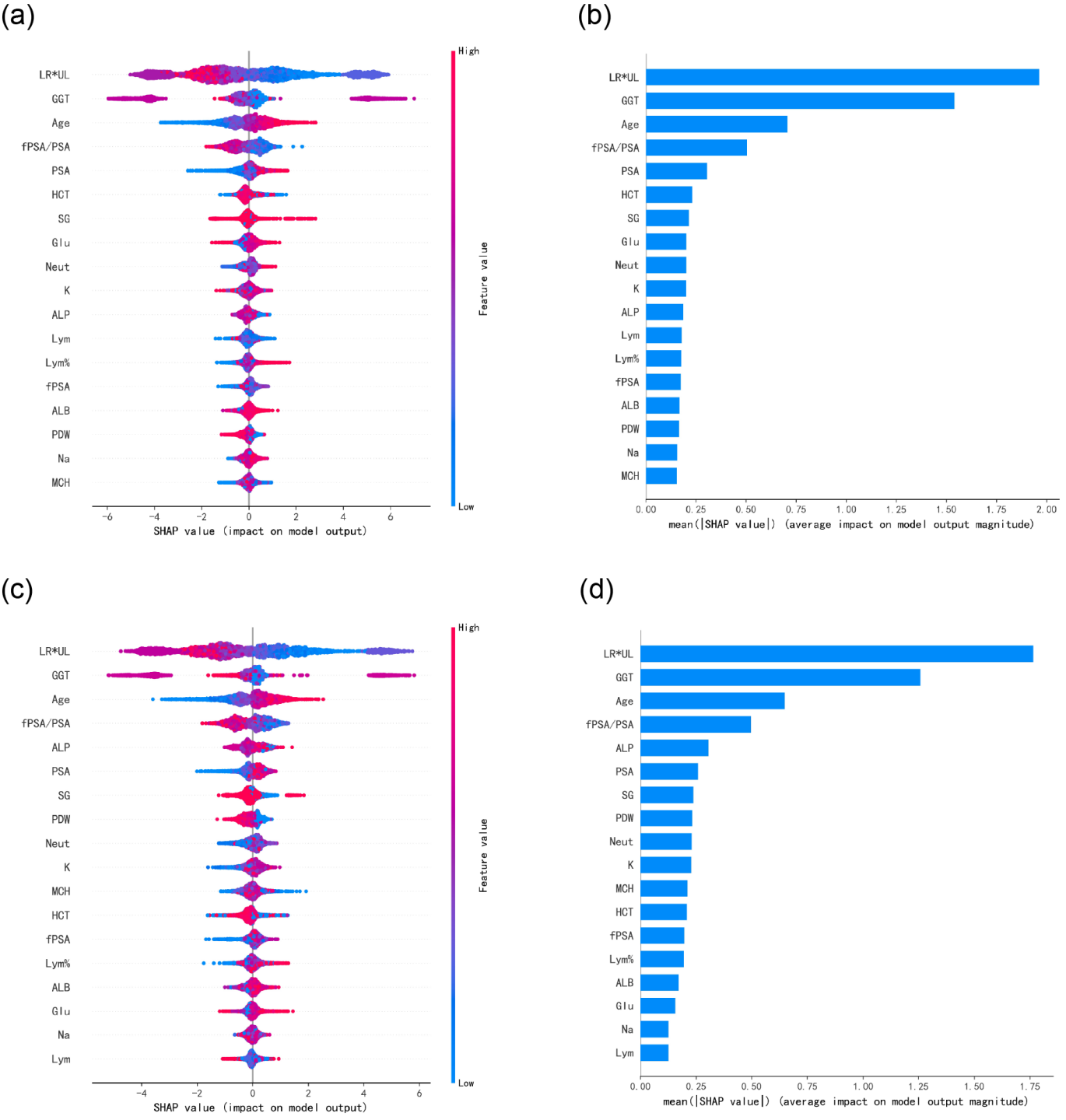


The SHAP value plotting the 18 most relevant features to predict the risk of PCa (a and b for PSA 4.0-20.0ng/ml; c and d for PSA 4.0-10.0ng/ml). On the X-axis, each feature’s contribution is shown. A feature with a negative Shapley value will favorably impact the prediction (decrease the risk of dying). The Y-axis indicated the value of the feature itself, with a high value (in red) is associated with a positive Shapley value that was associated with increased risk of PCa, while a low value (in blue) was associated with decreased Shapley value and the risk of PCa. SHAP, Shapley Additive exPlanations).

**Supplementary Figure 6. Violin plot of APCA scores distribution across** **different cohorts for two PSA ranges.**
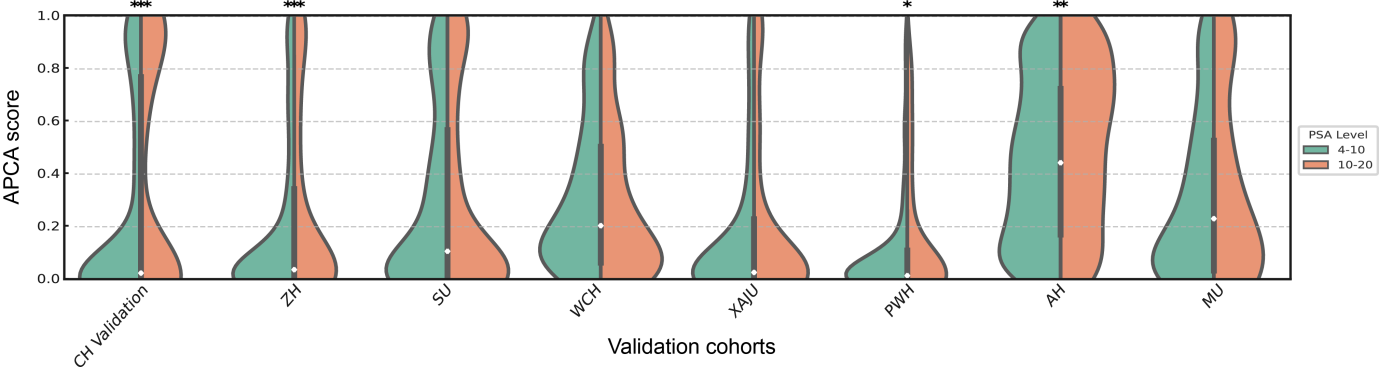


The plot illustrated the distribution of APCA scores within two PSA ranges (4.0-10.0 and 10.0-20.0 ng/ml) across different centers. The x-axis represented the various centers, while the y-axis represented the APCA score. Each violin plot at a center is divided into two parts: the left side represents the range of PSA 4.0-10.0 ng/ml, and the right side represents the range of 10.0-20.0 ng/ml. The width of each part signifies the distribution of APCA scores within that center and PSA range.

# **Supplementary Figure 7. Stacked violin plot of feature distribution across each cohort.**


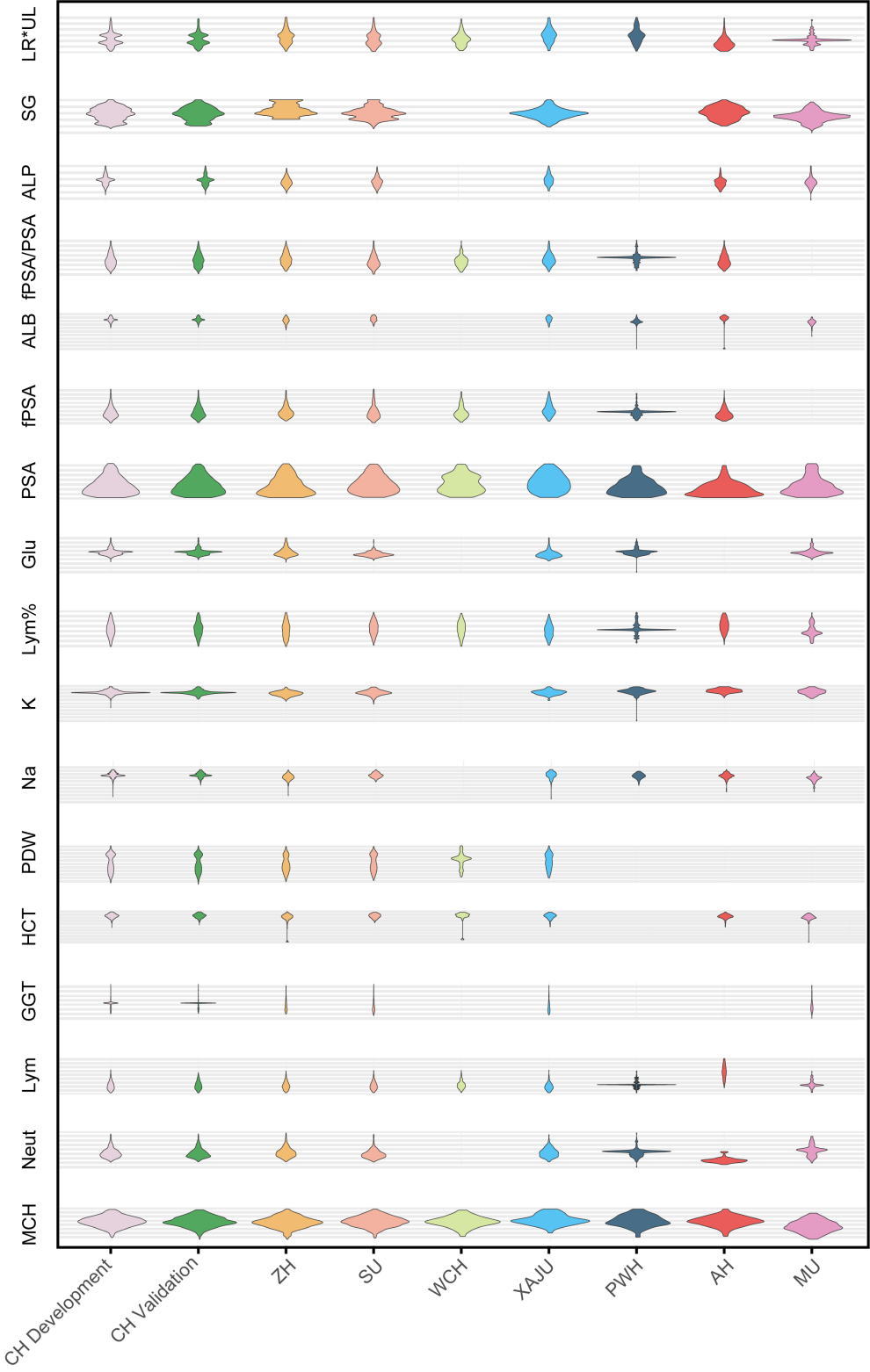


The stacked violin plot presents the distribution of 18 features across cohorts, after data imputation and the removal of the top and bottom 5% of extreme values for aesthetic purposes. Some features have been adjusted with non-proportional scaling for better visualization. The plot reveals a certain degree of heterogeneity in the features across different cohorts.

# **Supplementary Figure 8. Correlation matrix among different features and among different features and the APCA scores.**


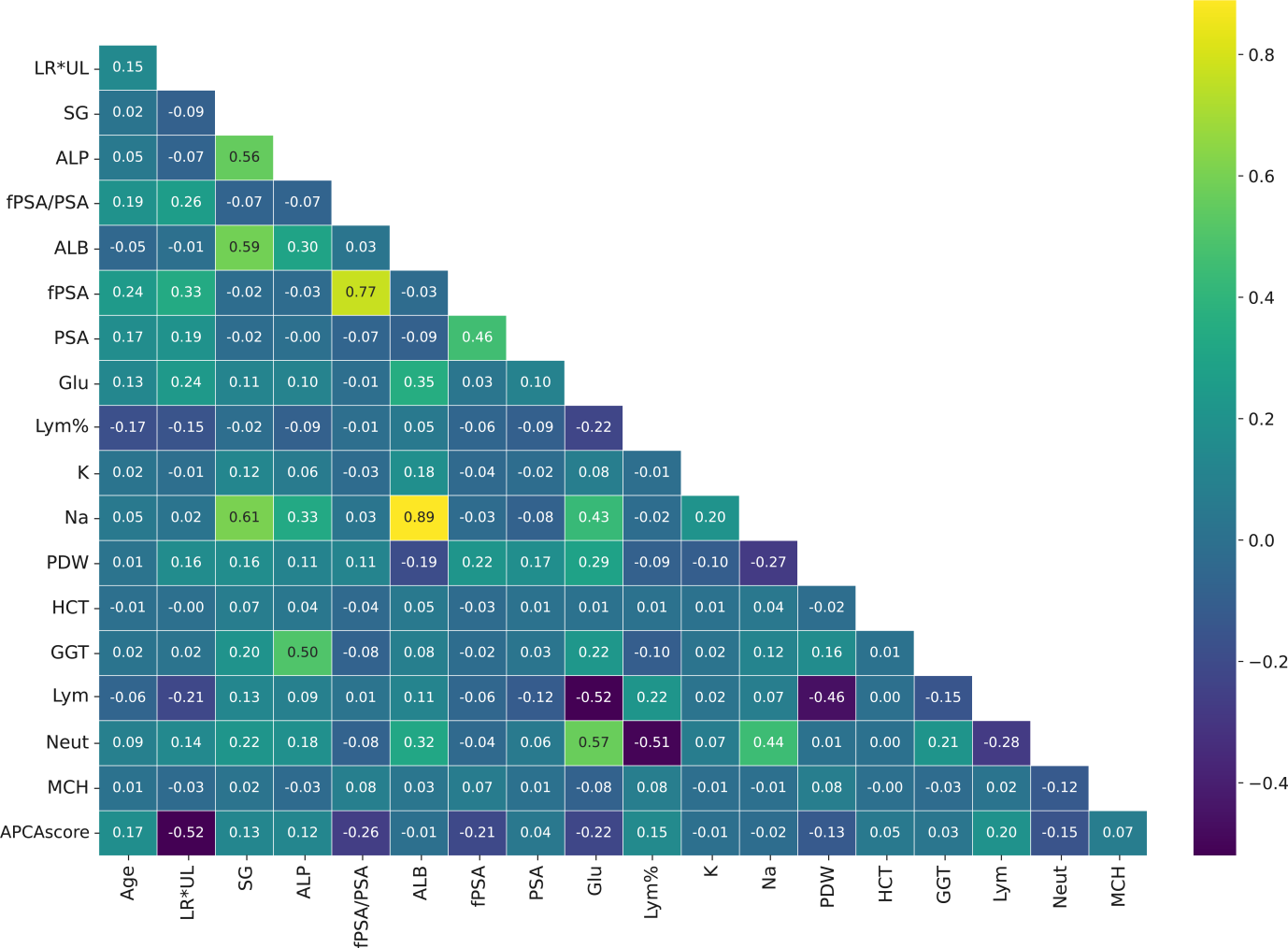


The correlation between different features, as well as their correlation with APCA scores were illustrated, using Spearman analysis.
